# Dual-ligand supramolecular nanofibers inspired by the renin-angiotensin system for the targeting and synergistic therapy of myocardial infarction

**DOI:** 10.7150/thno.53644

**Published:** 2021-01-27

**Authors:** Zhanpeng Wen, Jie Zhan, Hekai Li, Guanghui Xu, Shaodan Ma, Jianwu Zhang, Zehua Li, Caiwen Ou, Zhimou Yang, Yanbin Cai, Minsheng Chen

**Affiliations:** 1 Guangdong Provincial Biomedical Engineering Technology Research Center for Cardiovascular Disease, Department of Cardiology and Laboratory of Heart Center, Sino-Japanese Cooperation Platform for Translational Research in Heart Failure, Zhujiang Hospital, Southern Medical University, Guangzhou 510280, China.; 2 Shunde Hospital, Southern Medical University, the First People's Hospital of Shunde, Foshan 528300, China.; 3 School of Pharmaceutical Sciences, Southern Medical University, Guangzhou 510515, China.; 4 Department of Cardiology, Nanfang Hospital, Southern Medical University; Guangzhou 510515, China.; 5 Key Laboratory of Bioactive Materials, Ministry of Education, State Key Laboratory of Medicinal Chemical Biology, College of Life Sciences, Nankai University, Tianjin 300071, China.; 6 Jiangsu Center for the Collaboration and Innovation of Cancer Biotherapy, Cancer Institute, Xuzhou Medical University, Xuzhou 221004, Jiangsu, China.

**Keywords:** supramolecular self-assembly, myocardial infarction, renin-angiotensin system (RAS), targeted therapy, synergistic effect

## Abstract

**Rationale:** The compensatory activation of the renin-angiotensin system (RAS) after myocardial infarction (MI) plays a crucial role in the pathogenesis of heart failure. Most existing studies on this subject focus on mono- or dual-therapy of blocking the RAS, which exhibit limited efficacy and often causes serious adverse reactions. Few studies have been conducted on targeted therapy based on the activated RAS post-MI. Thus, the development of multiple-functional nanomedicine with concurrent targeting ability and synergistic therapeutic effect against RAS may show great promise in improving cardiac function post-MI.

**Methods:** We utilized a cooperative self-assembly strategy constructing supramolecular nanofibers— telmisartan-doped co-assembly nanofibers (***TDCNfs***) to counter-regulate RAS through targeted delivery and combined therapy. ***TDCNfs*** were prepared through serial steps of solvent exchange, heating incubation, gelation, centrifugation, and lyophilization, in which the telmisartan was doped in the self-assembly process of ***Ang1-7*** to obtain the co-assembly nanofibers wherein they act as both therapeutic agents and target-guide agents.

**Results: *TDCNfs*** exhibited the desired binding affinity to the two different receptors, AT1R and MasR. Through the dual ligand-receptor interactions to mediate the coincident downstream pathways, ***TDCNfs*** not only displayed favorably targeted properties to hypoxic cardiomyocytes, but also exerted synergistic therapeutic effects in apoptosis reduction, inflammatory response alleviation, and fibrosis inhibition *in vitro* and *in vivo*, significantly protecting cardiac function and mitigating post-MI adverse outcomes.

**Conclusion:** A dual-ligand nanoplatform was successfully developed to achieve targeted and synergistic therapy against cardiac deterioration post-MI. We envision that the integration of multiple therapeutic agents through supramolecular self-assembly would offer new insight for the systematic and targeted treatment of cardiovascular diseases.

## Introduction

Cardiovascular diseases (CVDs) are the largest cause of death worldwide 1. Myocardial infarction (MI), the most serious clinical manifestation of CVDs, poses an acute threat to global health and affects more than 7 million individuals every year 2. Prompt surgical procedures post-MI could help rescue the damaged myocardium by restoring arterial blood flow to the heart; however, a series of secondary left ventricular lesions occur when the compensatory mechanisms underlying the maintenance of hemodynamic stability and cardiac output are continuously activated, which could gradually lead to ventricular remodeling and ultimately lead to cardiac failure 3,4. Since the 5-year survival rate for patients with cardiac failure is only 50% 5, ameliorating the deterioration of cardiac function to prevent cardiac failure is essential for post-MI prognosis.

The renin-angiotensin system (RAS), a peptidergic system of enzymatic cascades for the homeostatic control of cardiovascular physiology, plays a central role in the pathogenesis and progression of heart failure 6. The overactivation of the RAS has been attributed to various adverse outcomes such as vasoconstriction, fibrosis, and cardiac remodeling, in which Ang II exerts key detrimental effects by binding to the Ang II type I receptor (AT1R). Thus, pharmacological antagonism of the RAS through AT1R blockers (ARB drugs) evolves as the cornerstone of clinical therapy for human heart failure 7. Even though the current ARB drugs have shown restricted efficacy in preventing the occurrence of adverse cardiac events, they have been proven to be ideal target ligands for lesions of overexpressed AT1R in multiple diseases because of their strong binding affinity to AT1R 8,9. On the other hand, several homologous bioactive peptides derived from angiotensinogen act as biased ligands for counter-regulatory RAS through highly diverse targets, which contributes to their cardioprotective benefits 10-12. A myriad of pharmacological pathways has already been exploited to demonstrate the individual treatment powers of these homologous peptides, promising them as novel drugs for alleviating cardiovascular diseases. Access of bioactive peptides to the clinic, however, is limited owing to their unfavorable pharmacokinetic defects, including extremely short half-life and untargeted delivery 13,14. Functionalization of vasoactive peptides to form more stable analogues or using a bispecific combination strategy (endow them with targeting units) are attractive approaches to promote their clinical applications.

Supramolecular self-assembly possesses significant potential for combination therapy as it involves the integration of multiple components into one single nanoplatform 15-17. Small molecular amphiphilic peptides are widely used to prepare supramolecular self-assembly nanostructures in nanomedicine 18-20, tissue engineering 21-23, regenerative medicine 24-26, and cell signaling studies 27-29 owing to their remarkable properties, including excellent biocompatibility, programmable primary structure, and easy availability. The interest in peptides-based nanomaterials primarily stems from their unique biological advantages (e.g., preferential binding affinity to proteins or direct mimicking of bioactive molecules) as well as the benefits these offer as nanocarriers (such as targeted delivery and high tissue permeability) 30-33. Through the incorporation of amphiphilic motifs, bioactive peptides can self-assemble into supramolecular nanostructures with enhanced tissue retention capacity and improved bioavailability 34,35. Additionally, multiple weak intermolecular interactions, including π-π stacking, hydrophobic interactions, and hydrogen bonds, could also be used to realize the nanoengineering of small molecular drugs. Several pioneering studies have demonstrated that the introduction of hydrophobic photosensitizers, near-infrared dyes, or anti-cancer drugs into self-assembly nanostructures can serve as functional components with multiple favorable therapeutic features 36-38.

**Scheme 1:**
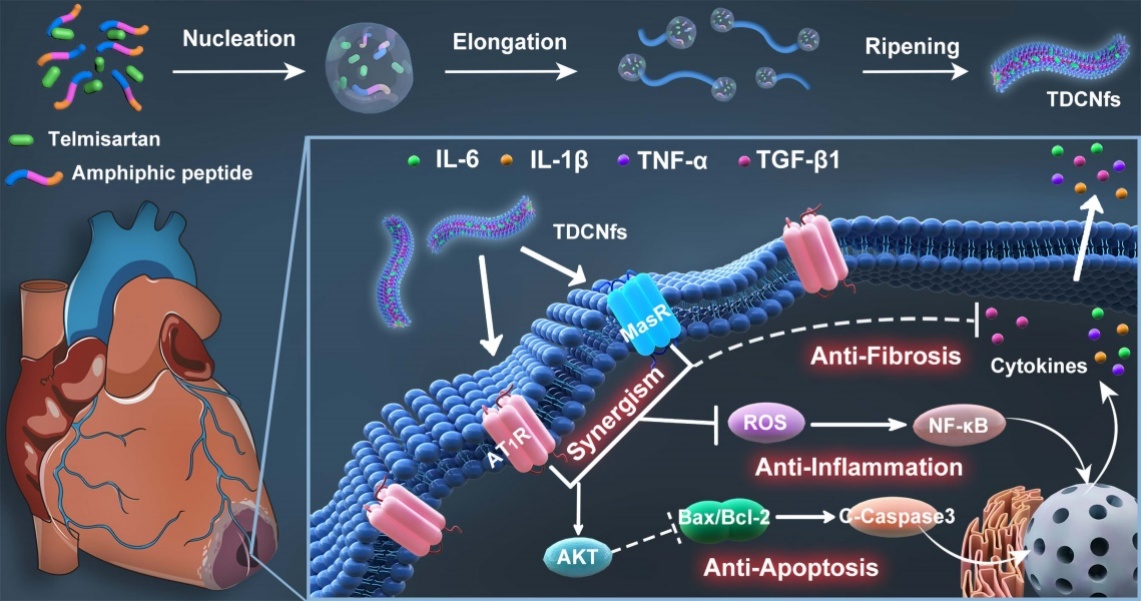
Dual-ligand supramolecular nanofibers formed by telmisartan-doped peptide self-assembly improve post-MI cardiac function through targeted and synergistic effects on counter-regulatory RAS.

In this study, we proposed a supramolecular nanofiber strategy based on dual ligands to counter-regulate RAS by target delivery and combined therapy. As shown in Scheme 1, we chose a supramolecular self-assembly motif as a building block to conjugate the therapeutic heptapeptide angiotensin 1-7 (***Ang 1-7***). Telmisartan (***Tel***), with the highest affinity to AT1R in ARBs due to the unique “delta lock” molecular structure 39, was employed as a target-guide ligand molecule to dope in the self-assembly process for the resulting ***Tel*** doped co-assembly nanofibers (***TDCNfs***). The two therapeutic components in the nano-platform exerted enhanced anti-apoptotic, anti-inflammatory, and anti-fibrotic effects. Owing to the high drug loading efficiency and synergistic effects in a continuous signaling cascade, ***TDCNfs*** showed great potential for novel targeted and combined medication to prevent cardiac failure in post-MI.

## Results and Discussion

### Molecule design and co-assembly behavior

**Figure 1:**
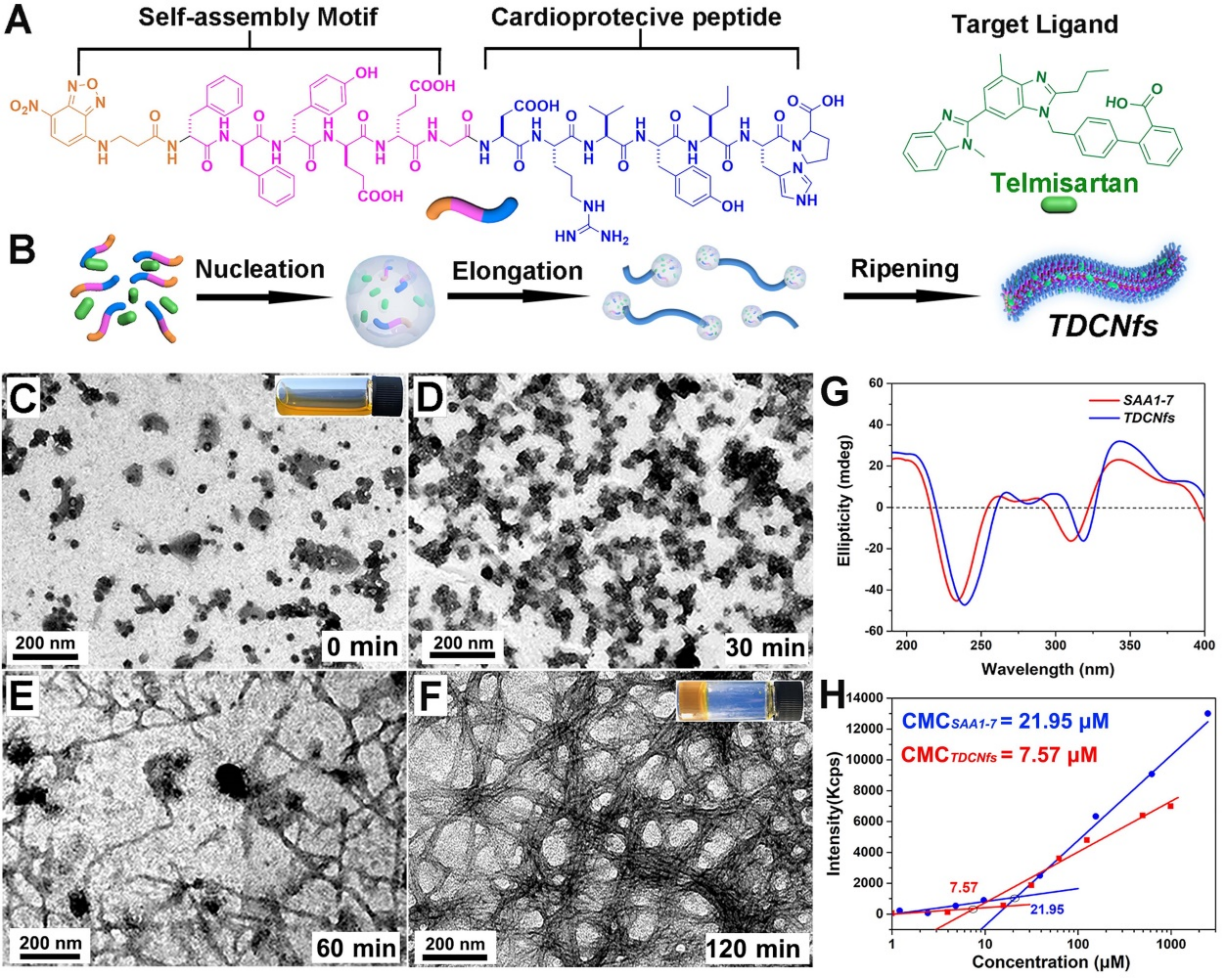
A) Molecular chemical structure of ***SAA1-7*** and ***Tel***; B) The classical nucleation-elongation mechanism to form*** TDCNfs***; C) - F) TEM images of different time points in the process of ***TDCNfs*** formation, the images inserted represented the initial solution and the final hydrogel; G) Circular dichroism of ***SAA1-7***and ***TDCNfs***; H) Critical micelle concentration of ***SAA1-7*** and ***TDCNfs****.*

To construct the dual-ligand supramolecular nanofibers, we designed and synthesized a peptide molecule NBD-^D^F^D^F^D^Y^D^E^D^EG-DRVYIHP (***SAA1-7***, Figure 1A & Figure S1) as a hydrogelator. NBD-^D^F^D^F^D^Y^D^E^D^EG was a potent self-assembly motif, in which the fluorophore NBD could be conducive to targeted identification, and the glutamic acid E was used to regulate the hydrophilicity of molecule preventing it from precipitation 40,41; the substitutive D-amino acid was introduced to improve the *in vivo* stability of the molecule as the organisms without a protease capable of its degradation 42; the vasoactive peptide ***Ang1-7*** (DRVYIHP) possessed a biased activity toward AT1R as well as agonism on multiple other receptors (mainly acting on the ACE2/MasR axis) for counter-regulatory of RAS in many cardiovascular diseases 43. ***SAA1-7*** could self-assemble into a transparent hydrogel in PBS (pH 7.4) at a concentration of 1.0 wt% *via* a simple heat-cooling process, in which we observed uniform nanofibers with diameters of 7.6 ± 1.8 nm using transmission electron microscopy (TEM) (Figure S2). Its corresponding rheological properties are shown in Figure S3. The drug loading efficiency and the stability of nanocarriers are attributed to the affinity between amphiphilic peptides and hydrophobic molecules. Accordingly, we screened the optimal candidate from five clinical drugs (losartan, valsartan, irbesartan, telmisartan, and candesartan) based on their affinity toward ***SAA1-7*** by autodock (Figure S4 & Table S1). The results indicated that ***Tel***is an ideal model molecule for the construction of nanomedicine. We then prepared ***Tel***-doped co-assembly nanofibers (***TDCNfs***) through a series of steps including solvent exchange, heating incubation, hydrogelation, centrifugation, and lyophilization (Figure S5). The molar ratio of ***Tel*** affected the process of hydrogelation, in which 50% of ***Tel*** was proved to be the maximal proportion to maintain the hydrogel (Figure S6). This result was consistent with the rheological properties of ***TDCNfs***with various molar concentrations of telmisartan (Figure S7). We measured the encapsulation efficiency (EE) and drug loading (DL) of ***TDCNfs***. As the molar ratio of ***Tel***increased, the EE and DL would increase to favorable values of 49.12% and 10.54%, respectively, as long as the stable hydrogel formed. Once the ***Tel*** reached 70%, the EE and DL values decreased sharply and approached approximately zero owing to the collapse of the hydrogel (Figure S8).

Subsequently, we studied the co-assembly behaviors and the underlying mechanism of ***TDCNfs***. Because amphiphilic and hydrophobic molecules would rapidly precipitate during solvent conversion and aggregate into nanoparticles, we speculated that the co-assembly of hydrophobic ***Tel*** and amphiphilic peptides followed classical nucleation-dependent polymerization to form supramolecular nanofibers, consisting of an initial nucleation event and the subsequent growth of fibrillary structure (Figure 1B) 44. We monitored and compared the nanostructures in the process of hydrogelation at different time points by TEM. At the beginning, unevenly clustered particles were distributed randomly (Figure 1C); 30 minutes after heat-cooling, the nanoparticles increased and became susceptible to merging as an evidence of self-assembly proceeding (Figure 1D); 60 minutes later, the nanoparticles accumulated into larger-sized clusters by fusion as a cluster-core, in which the nanofibers formed and elongated around the core (Figure 1E); eventually, the network of dense nanofibers with diameters of 13.2 ± 2.3 nm was generated in the final hydrogel at 120 min (Figure 1F). These results were consistent with our assumption of the molecular mechanism underlying the formation of ***TDCNfs***.

As a helpful tool to investigate the molecular arrangements and driven forces of self-assembly, circular dichroism (CD) was performed to assess the superstructures of ***SAA1-7***and ***TDCNfs***. As shown in Figure 1G, they both shared similar features of a peak near 190 nm (π-π* transition) and a trough near 220 nm (n-π* transition) represented the π-π stacking of aromatic units, which suggested that their backbone adopted the common β-sheet configurations 45. The nanofibers of ***SAA1-7*** and ***TDCNfs*** were also examined using Fourier transform infrared spectroscopy (FT-IR) to further explore the H-bonding-assisted self-assembly and conformations. Amide I and II bands clearly appeared around 1650 cm^-1^ and 1550 cm^-1^, respectively, which were in close agreement with amine N-H stretching and NH_2_ vibration mode. All amide I bands were assigned to a β-sheet structure (1637-1613 cm^-1^) in the presence of plentiful hydrogen bonding (3200-3500 cm^-1^) (Figure S9) 46. The fluorescent spectra offered useful information regarding the interaction of aromatic rings, as shown in Figure S10. The emission peak of the ***SAA1-7*** solution was centered at 303 nm, whereas the peaks underwent a red-shift to asymmetric peaks with a maximum at 329 nm for the ***SAA1-7*** gel and 330 nm for ***TDCNfs***, supporting the evidence of π-π stacking for self-assembly 47. Compared with the ***SAA1-7***gel, the broad shoulders of ***TDCNfs***further indicated stronger aromatic-aromatic interactions between the aromatic residues of peptide and ***Tel*** in the structural evolution process. The critical micelle concentration (CMC) of ***SAA1-7*** and ***TDCNfs***decreased from 21.95 to 7.57 μM, indicating that ***Tel*** doping enabled the ***TDCNfs*** to assemble stably in aqueous solution at a lower concentration (Figure 1H). Moreover, ***TDCNfs***exhibited long-term storage stability due to the incorporation of D-amino acids that resist proteinase digestion. We observed that the colloidal solution of ***TDCNfs*** remained stable for up to 1 month without the separation of discernible precipitates or agglomerates in PBS (pH 7.4), which was verified by TEM (Figure S11). The resulting release profile of ***Tel*** demonstrated that the proportion of ***SAA1-7*** was not cleaved by proteinase exceeding 45% after 24 h. The release of ***Tel*** from ***TDCNfs*** was initiated when ***SAA1-7*** was proteolyzed and lasted for more than 24 h, indicating that ***TDCNfs'*** moderate resistivity against enzymatic digestion could be favorable to prolong its circulation time *in vivo* (Figure S12).

### Bioactivity and targeted ability of *TDCNfs*

Primary neonatal rat cardiomyocytes (NRCMs) and neonatal rat cardiac fibroblasts (NRCFs) were cultured with gradient concentrations of ***TDCNfs***. The results revealed that the viability of cells increased with the concentration of ***TDCNfs*** ranging from 0.01 to 10 μM, indicating that ***TDCNfs*** had a stimulatory effect. However, treatment with 100 μM of ***TDCNfs*** decreased the viability of cells, suggesting that the positive stimulatory effect of ***TDCNfs*** on cells was exerted only within a limited range of concentrations (Figure S13-14).

**Figure 2:**
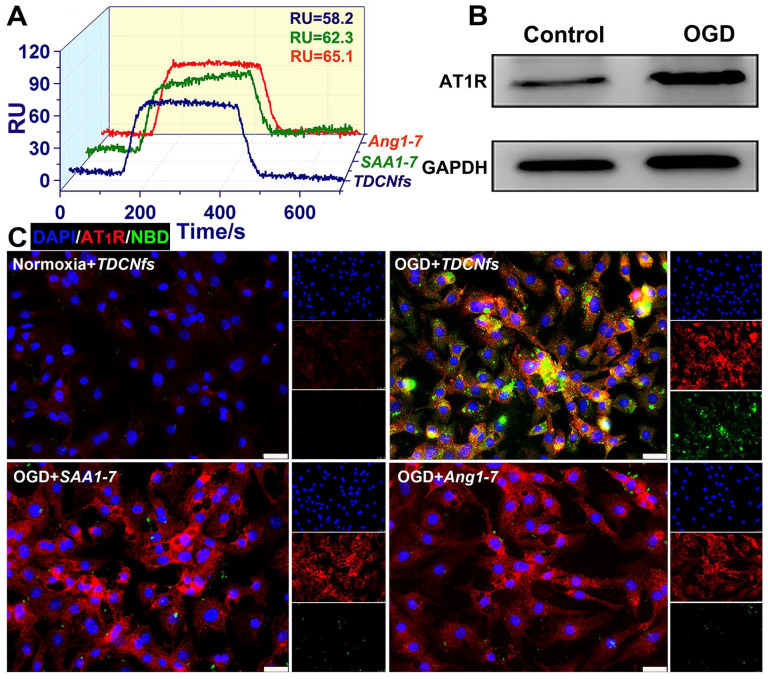
A) Evaluation of the binding affinity of ***Ang1-7***, ***SAA1-7***, ***TDCNfs*** to MasR; B) Western blot of AT1R in NRCMs under normoxia or OGD conditions, GAPDH as the internal reference; C) Co-localization of AT1R (red) with ***^NBD^Ang1-7***, ***^NBD^SAA1-7***, and ***TDCNfs*** (green) in NRCMs under normoxia or OGD conditions; scale bar = 25 µm.

An orphan receptor Mas (MasR) was identified for the predominantly activated unit of ***Ang1-7*** to exert vasodilatory and cardioprotective effects; therefore, we used surface plasmon resonance imaging (SPRi) to determine the binding affinity of ***TDCNfs*** to recombinant human MasR (rhMasR) 48. First, rhMasR was immobilized on the surface of a sensor chip, then ***Ang1-7***, ***SAA1-7***, and ***TDCNfs*** were diluted in PBS at concentrations ranging from 0.001 μM to 10 μM for kinetics tests and affinity assessment. The ***Ang1-7*** exhibited a K_D_ Value of 10.3 nM to rhMasR, whereas the K_D_ value of ***SAA1-7***, and ***TDCNfs*** was 12.6 nM and 16.5 nM (Figure S15). Their binding signals were shown in the SPR response images, and the obtained binding values (RU) of the three compounds were 65.1 (***Ang1-7***), 62.3 (***SAA1-7***), and 58.2 (***TDCNfs***), respectively, which indicated that their binding affinities are approximately similar to MasR (Figure 2A).

AT1R was overexpressed in NRCMs under hypoxic environments, which was confirmed in an oxygen/glucose-deprived (OGD) model that simulated the abovementioned conditions (Figure 2B & Figure S16) 49. Accordingly, we assumed that the affinity between ***Tel*** and AT1R could improve the targeting ability of ***TDCNfs***. ***Ang1-7*** was endowed with fluorescence similar to that of ***SAA1-7***and ***TDCNfs*** by NBD labeling (***^NBD^Ang1-7***, Figure S17). We compared the uptake behaviors of different compounds on NRCMs by FACS. A significant increase in the internalization of ***^NBD^Ang1-7***, ***SAA1-7***, and ***TDCNfs*** in the OGD condition was observed than that of ***TDCNfs*** under normoxic conditions 3.28-fold and 3.49-fold higher uptake of ***TDCNfs*** was calculated when compared to that of ***SAA1-7*** and ***^NBD^Ang1-7***, respectively (Figure S18). The fluorescent co-localization of them with AT1R was also studied by confocal microscopy. As shown in Figure 2C, NRCMs under OGD conditions expressed high levels of AT1R (red fluorescence), which was in accordance with the above results. A slightly overlapped signal of ***^NBD^Ang1-7*** and ***SAA1-7***with AT1R could be visualized on OGD-conditioned NRCMs. In contrast, elevated co-localization of ***TDCNfs*** with AT1R was mainly distributed on the plasma membrane. When the cells were pre-saturated with free ***Tel*** at a concentration of 0.2 mM and 2 mM, the fluorescent signal of ***TDCNfs*** decreased significantly (Figure S19). Collectively, ***TDCNfs*** were found to have the strongest affinity with AT1R among these three compounds, while the existence of ***Tel*** in ***TDCNfs*** might facilitate AT1R targeting.

### Anti-apoptotic studies

**Figure 3:**
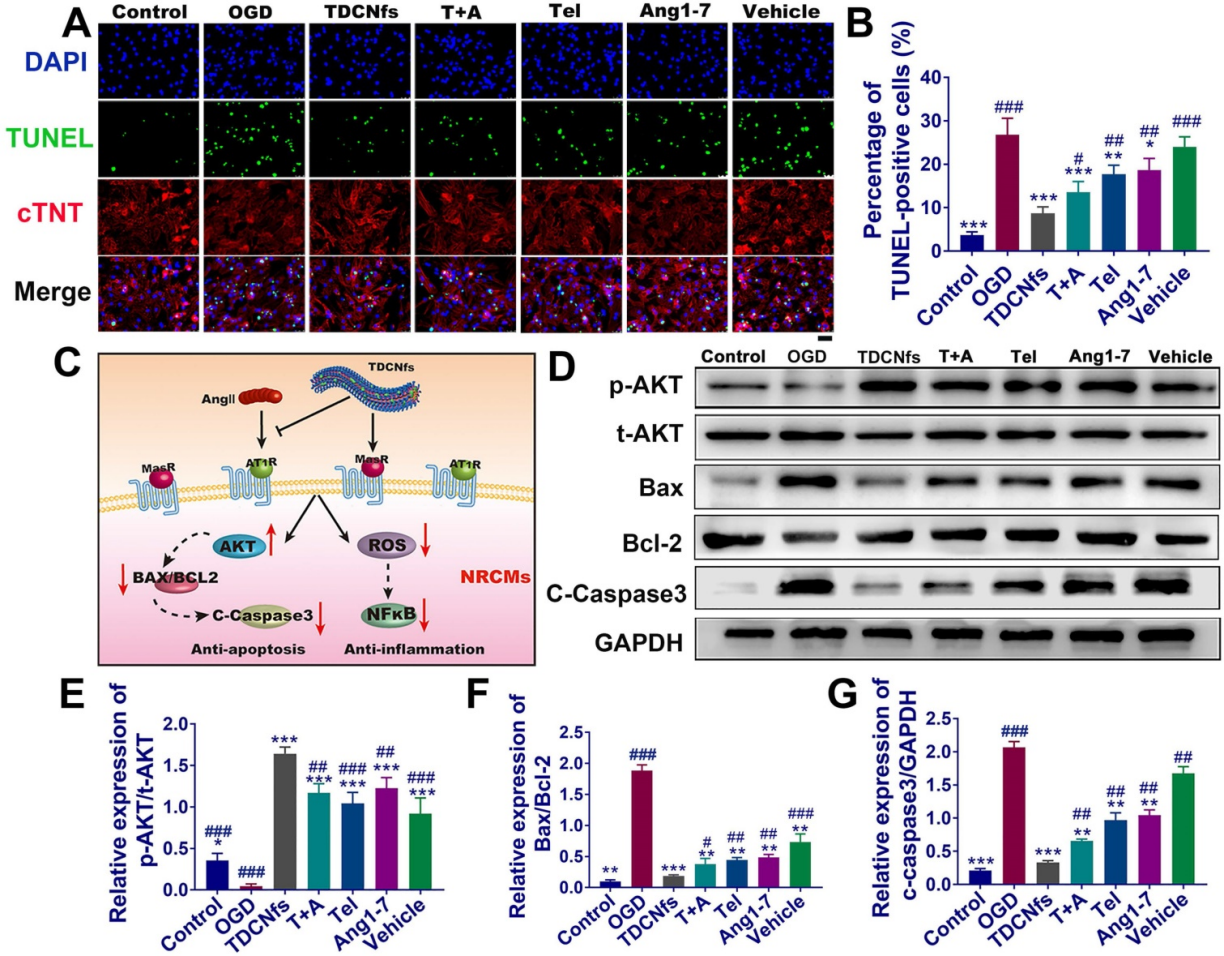
A) TUNEL and cTnT (cardiomyocyte specific marker) staining of NRCMs in different groups; B) Quantitative analysis of TUNEL-positive NRCMs; C) Illustration of the underlying signaling pathways for ***TDCNfs'*** cardioprotective actions; D) Expression of apoptosis-related proteins measured in western blotting, GAPDH as an internal reference; E)-G) Quantitative analysis of protein bands in D based on optical density. Scale bar = 50 µm, ^*^p < 0.05 *vs.* OGD, ^**^p < 0.01 *vs.* OGD, ^***^p < 0.001 *vs.* OGD, ^#^*p* < 0.05 *vs.* ***TDCNfs***, ^##^*p* < 0.01 *vs.* ***TDCNfs***, ^###^*p* < 0.001 *vs.* ***TDCNfs***, n = 3.

The apoptosis of cardiomyocytes under hypoxia is an important factor leading to myocardial damage and cardiac failure 50. The hypoxia damage of NRCMs was minimized upon treatment with 10 μM ***TDCNfs***(Figure S20A), which was coincident with the cytocompatibility pattern stated above. This further encouraged us to study its potential in anti-apoptotic and associated mechanisms. First, a scrambled sequence of ***Ang1-7*** (YRVIPHD) was incorporated with the self-assembly motif to obtain a control molecule as ***vehicle***(Figure S21). Apoptosis of OGD NRCMs was evaluated in the following groups: ***TDCNfs***, ***Tel*** plus ***Ang1-7*** (***T+A***), ***Tel***, ***Ang1-7***, ***vehicle***, and a blank group named OGD (subsequent experiments adopted this grouping rule without specific notation). Among all groups, ***TDCNfs*** treatment exerted an optimal protective effect on NRCMs with an improvement of up to 1.22-fold compared to the OGD group (Figure S20B). TUNEL staining was then performed to evaluate the apoptosis of NRCMs (stained with cTNT). The enumeration of TUNEL-positive cells indicated that 26.70% of NRCMs in the OGD group underwent apoptosis. In contrast, this proportion decreased to 8.30%, 13.15%, 17.62% 18.96%, and 23.5% in the ***TDCNfs***, ***T+A***, ***Tel***, ***Ang1-7***, and ***vehicle*** groups, respectively (Figure 3A-B), implying that ***TDCNfs*** exhibited the highest anti-apoptotic potential.

As illustrated in Figure 3C, although ***TDCNfs*** could interact with different receptors based on dual ligands, some studies reported that they could impart the cardioprotective effects by actuating the consistent downstream signaling pathway to counter-regulatory RAS 12. We first investigated the phosphorylation of AKT protein, which plays an important role in cell survival 51. The expression of p-AKT in NRCMs was inhibited in the OGD group, but it was significantly enhanced in the ***TDCNfs***, ***T***+***A***, ***Tel***, ***Ang1-7***, and ***vehicle*** groups (all increased more than 10-fold (Figure 3D-E), indicating that the intervention by different groups of compounds could ameliorate the deteriorative cell survival under hypoxia. The *BCL2* family associated with the activation of the caspase cascade stood for the intrinsic pathway of apoptosis, in which the ratio of Bax/Bcl-2 expression level was generally used to evaluate the progression of apoptosis. Compared to the control group, the Bax/Bcl-2 value of NRCMs in OGD increased by 19.63-fold. After treatment with ***TDCNfs***, ***T+A***, ***Tel***, ***Ang1-7***, and ***vehicle***, the values decreased by 90.2%, 79.9%, 76.3%, 74.2%, and 67.7%, respectively. The expression of cleaved caspase-3 in different groups was fully compliant with the tendency of Bax/Bcl-2 ratios, and the lowest expression was observed in the ***TDCNfs*** group (Figure 3D, F-G). These results above demonstrated that ***TDCNfs*** exhibited a synergistic anti-apoptotic effect by regulating apoptosis-associated proteins.

### Reactive oxygen species (ROS) and inflammation studies

**Figure 4:**
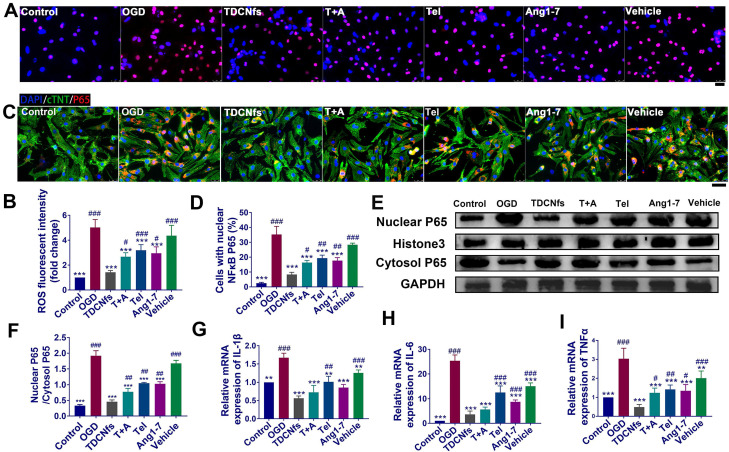
A) ROS level detection of NRCMs in different groups using DHE probe (red) ; B) Quantification of the DHE fluorescent intensity in A; C) Immunofluorescence assay for the nuclear transport of NF-κB (p65) in different groups; D) Quantification of the percentage of cells with nuclear P65 in C; E) Western blot of NF-κB (p65) from nuclear and cytosol, Histone 3 and GAPDH serve as an internal reference; F) Quantification of protein bands in E using densitometry; G) - I) The levels of IL-1β, IL-6, and TNF-α in different groups determined by RT-qPCR. Scale bar = 25 µm. ^**^p < 0.01 *vs.* OGD, ^***^p < 0.001 *vs.* OGD, ^#^*p* < 0.05 *vs. **TDCNfs***, ^##^*p* < 0.01 *vs. **TDCNfs***,^ ###^*p* < 0.001 *vs. **TDCNfs***, n = 3.

The increase in oxidative stress and inflammation plays critical roles in cardiomyocyte death and post-MI pathological alterations 52. We first employed the DHE probe to detect the expression of superoxide anions to evaluate the levels of ROS in NRCMs. As shown in Figure 4A, the highest number of red fluorescent dots (red) was observed in the OGD group, implying the ROS level in these NRCMs was significantly higher. The statistical results demonstrated that the fluorescence intensity of superoxide anion in the OGD group increased 5.72-fold compared to that in the control group. This value was altered to 1.50-, 2.72-, 3.23-, 3.01-, and 4.44-fold upon treatment with ***TDCNfs***, ***T+A***, ***Tel***,*** Ang1-7***, and ***vehicle***, respectively (Figure 4B), which showed that ***TDCNfs*** maximally inhibited the generation of ROS in NRCMs under simulated ischemia.

The level of ROS is closely related to the activation of the NF-κB pathway along with the release of inflammatory factors, which could further exacerbate cell damage and ROS generation repeatedly to form a vicious circle 53. Immunofluorescence analysis showed that OGD significantly promoted the translocation of p65, indicating activation of the NF-κB pathway. Nuclear transport of NF-κB was suppressed by varying degrees in different treatment groups. Particularly, NF-κB in the ***TDCNfs*** group was similar to that in the control group (Figure 4C-D). Consistent results of western blot are presented in Figure 4E. We performed a western blot assay to analyze p65 protein expression in both the cytoplasm and nucleus. OGD markedly increased p65 nuclear translocation, which was decreased by approximately 72.79% upon treatment with ***TDCNfs***. These results suggested that ***TDCNfs*** exhibited superior potential as regulators of the NF-κB pathway (Figure 4F).

We then assessed the levels of pro-inflammatory cytokines in different groups using RT-qPCR. As shown in Figure 4G-I, the expression of cytokine mRNA, including *IL1B*, *IL6*, and *TNFA* mRNA in the OGD group, increased by 1.67-, 25.38-, and 3.03-fold, respectively, which were significantly higher than those in the control group, respectively. ***TDCNfs***alleviated the expression of inflammatory factors more significantly than the other treatment agents did, only with 0.56-fold of IL-1β, 3.65-fold of IL-6, and 0.48-fold of TNF-α compared with the control. The results stated above suggest that ***TDCNfs*** effectively reduced the levels of ROS in OGD-conditioned NRCMs and prevented the activation of the NF-κB pathway to suppress the expression of inflammatory cytokines.

### Differentiation and migration of cardiac fibroblasts *in vitro*

**Figure 5:**
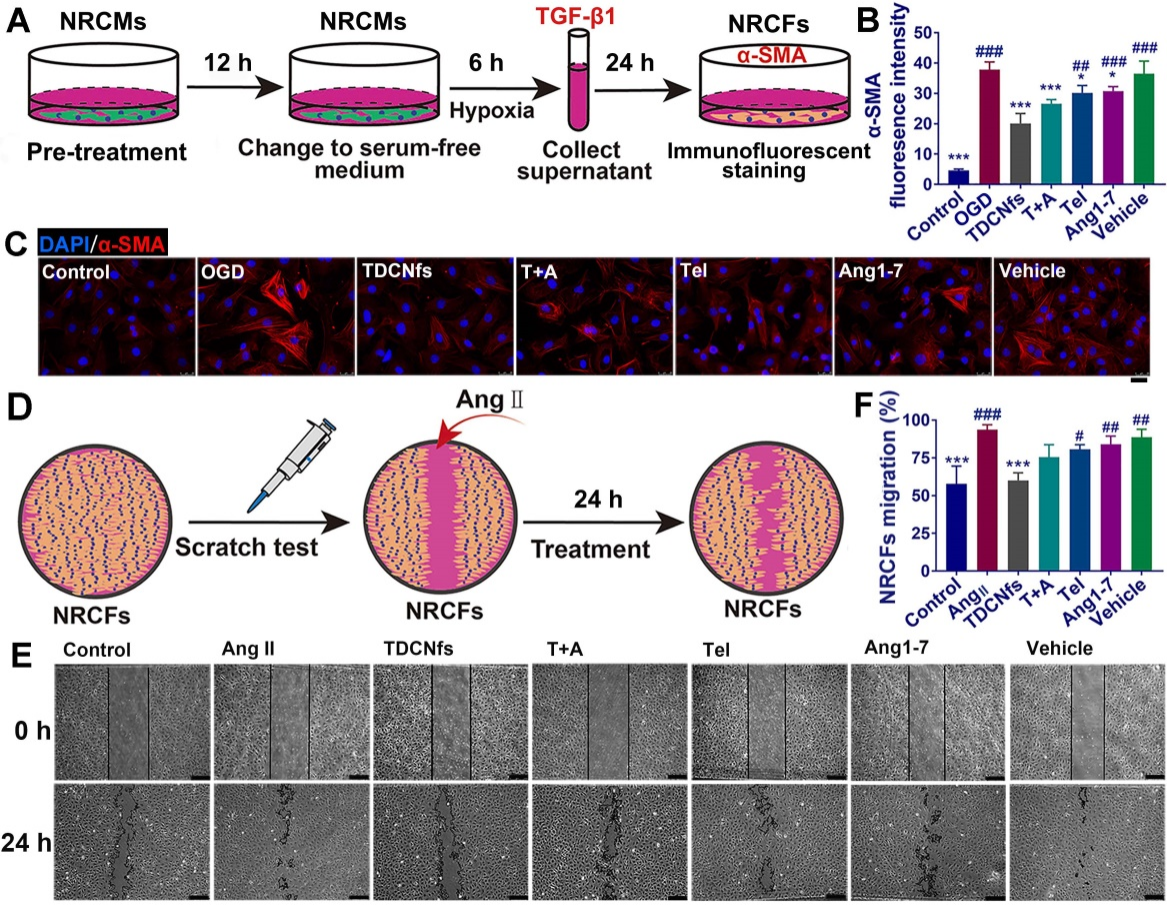
A) Scheme of conditioned medium interference experiments; B) immunofluorescence staining of α-SMA in NRCFs incubated with conditioned medium; C) Quantification of the immunofluorescence intensity in B; D) Scheme of the scratch test in NRCFs; E) Representative images of the scratch test in NRCFs at 0 h and 24 h under stimulation with Ang II (100 nM) in different groups; F) Quantification of NRCFs migration in E. Scale bar = 25 µm. ^*^p < 0.05 *vs.* OGD/Ang II, ^***^p < 0.001 *vs.* OGD/Ang II, ^#^*p* < 0.05 *vs. **TDCNfs***, ^##^*p* < 0.01 *vs. **TDCNfs***,^ ###^*p* < 0.001 *vs. **TDCNfs***, n = 3.

Recent studies had demonstrated that “stressed” cardiomyocytes signaled fibroblasts through paracrine factors, and consequently activated fibroblasts for initiating myo-differentiation and migration to drive myocardial fibrosis 54,55. To assess the effect of ***TDCNfs*** on the attenuation of pro-fibrotic paracrine factors, we first pretreated NRCMs with different drugs for 12 h, and then exchanged the medium with serum-free medium to further culture the cells for 6 h under OGD conditions (Figure 5A). As a major pro-fibrogenic cytokine derived from cardiomyocytes, the levels of TGF-β1 in the supernatant of the final medium were measured, and it was revealed that ***TDCNfs*** could inhibit the expression of TGF-β1 more effectively than other agents (Figure S22). Subsequently, we used the collected supernatant as conditioned medium to culture the NRCFs and quantified the expression of α-SMA to assess the degree of myofibroblast formation using immunofluorescence techniques. The quantified analysis of fluorescence indicated that pretreatment with ***TDCNfs*** reduced the levels of α-SMA in NRCFs by 46.98% compared to that in the OGD group. However, this value was only 32.79%, 20.48%, 16.98%, and 9.07% in groups treated with ***T+A***, ***Tel***, ***Ang1-7***, and ***vehicle***, respectively (Figure 5B-C).

Further investigations were conducted to explore the effect of ***TDCNfs*** on the resistance of NRCFs toward angiotensin II (Ang II, another pro-fibrosis factor) 56. We performed the scratch test to measure the migration of NRCFs as an evaluable indicator of fibrosis (Figure 5D). The results revealed that Ang II stimulation significantly increased the migration area of fibroblasts, and the mobility of NRCFs was inhibited to varying degrees by different drugs. Among them, ***TDCNfs*** presented a significant inhibiting effect on the NRCFs (Figure 5E-F). These results implied that ***TDCNfs*** exerted desirable anti-fibrogenetic effects *in vitro*.

### Targeting effect and distribution of *TDCNfs in vivo*

**Figure 6:**
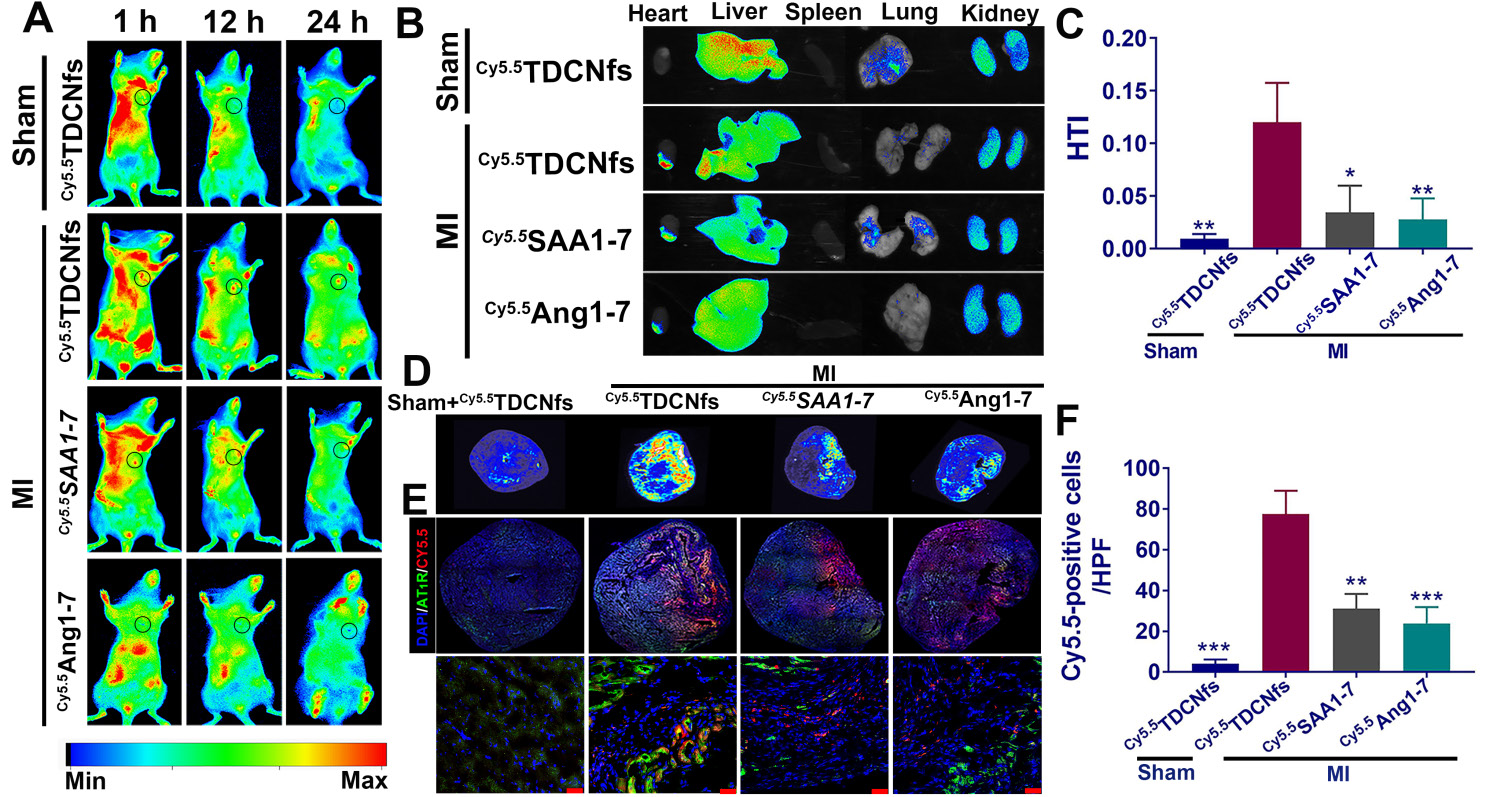
A) Fluorescence images of***^Cy5.5^ TDCNfs***, ***^Cy5.5^ SAA1-7***, ***^Cy5.5^ Ang1-7*** distribution in MI mice *in vivo* at predetermined time intervals. The black circles represent the position of the heart; B) Hearts and major organs dissected for *ex vivo* examination of the fluorescence signals; C) Quantification of the results described in B using heart-targeting index (HTI, calculated by heart fluorescence emission/liver fluorescence emission); D) Full cross-section of the hearts depicting distribution of the Cy5.5 labeled drugs. E) Co-localization signals of AT1R (green) and Cy5.5 labeled drugs (red), scale bars = 25 µm (enlarged images); F) Quantification of Cy5.5-positive cells in E. ^*^ *p* < 0.05 *vs.* MI+***^ Cy5.5^ TDCNfs***, ^**^ p < 0.01 *vs.* MI+***^ Cy5.5^ TDCNfs***, ^***^ *p* < 0.001 *vs.* MI+***^ Cy5.5^ TDCNfs***, n = 5.

The leaky vasculature after acute MI was conducive to the accumulation of targeted nanomaterials in the heart through enhanced permeability and retention (EPR) effect, although this passive uptake only occurred for a short time, which was insufficient for effective cardiac repair 57. The active targeting of AT1R by ***Tel*** increased the possibility of a valid cardioprotective action by*** TDCNfs***. To evaluate whether ***TDCNfs*** could accumulate in the infarcted area of the heart where AT1R was overexpressed (Figure S23), NBD was replaced with the near-infrared dye Cy5.5 to label the self-assembly molecule *in vivo* (***^Cy5.5^SAA1-7***, Figure S24).***^ Cy5.5^SAA1-7***could also form a hydrogel and subsequently form ***^Cy5.5^TDCNfs*** (Figure S25). A rheology test of***^Cy5.5^ TDCNfs*** was performed (Figure S26). Additionally, Cy5.5-labeled ***Ang1-7*** was synthesized as a control (***^Cy5.5^Ang1-7***, Figure S27). Following the ligation of the left anterior descending coronary artery (LAD) in C57BL/6 mice to construct the MI model, ***^Cy5.5^TDCNfs***, ***^Cy5.5^SAA1-7***, and ***^Cy5.5^Ang1-7*** were injected intravenously for *in vivo* imaging. As shown in Figure 6A, ***^Cy5.5^TDCNfs*** were barely detected in the Sham group at 1 h to 24 h, possibly because the integrity of the vascular endothelium made it impossible to penetrate. In comparison, the mice treated with ***^Cy5.5^TDCNfs*** exhibited stronger fluorescent signals in the heart than those treated with the other two agents, wherein the ***^Cy5.5^TDCNfs*** signal was retained for as long as 24 h. Notably, a relatively weak and unstable fluorescence signal was observed in mice injected with ***^Cy5.5^SAA1-7***, possibly owing to the EPR effect in the injured vasculature in the ischemic heart, which might have led to the penetration and short-term retention of nanofibers.

The heart and other major organs were harvested 24 h post-injection for *ex vivo* imaging. As shown in Figure 6B, the hearts from mice belonging to the MI group treated with ***^Cy5.5^TDCNfs*** displayed a stronger fluorescent signal than those injected with^***Cy5.5***^***SAA1-7*** or ***^Cy5.5^Ang1-7***. Moreover, the signal in the infarcted heart was markedly higher than that in the non-infarcted heart, even when both were treated with ***^Cy5.5^TDCNfs***, which confirmed the greater localization of ***^Cy5.5^TDCNfs*** to the infarcted area. Apart from the hearts, the fluorescence from the three Cy5.5-labeled agents was primarily detected in the liver and kidneys, which suggested that the compounds were excreted through the major metabolic organs. Quantitative analysis was conducted to assess the targeting ability using the heart-targeting index (HTI, calculated by heart fluorescence emission/liver fluorescence emission). Figure 6C shows that the HTI value in the MI+^***Cy5.5***^***TDCNfs*** group was 2.6- and 3.3-fold higher than those in the ***^Cy5.5^SAA1-7***and ***^Cy5.5^Ang1-7*** groups, and was also 10-fold higher than that in the Sham group, which confirmed the distinctive targeting potential of ***^Cy5.5^TDCNfs*** toward the infarcted heart.

To clearly observe the drug distribution, a near-infrared laser imager was used to capture the images of hearts in full cross-sections among different groups (Figure 6D). As the results show, MI+***^Cy5.5^TDCNfs***exhibited stronger fluorescent signals in the infarcted site than in the other groups, which is consistent with the aforementioned conclusion. Further experiments were performed to explain the interaction between AT1R and ***^Cy5.5^TDCNfs*** at the infarcted site. Immunofluorescence assay was employed to stain with an anti-AT1R antibody in the samples across the infarct zone. As shown in Figure 6E-F, co-localization signals of AT1R (green) and ***^Cy5.5^TDCNfs*** (red) appeared in the tissue sections, while the red fluorescence was negligible in the non-infarcted heart. As expected, the overlapping signals of ***^Cy5.5^SAA1-7*** or ***^Cy5.5^Ang1-7*** group were significantly lower than those of ***^Cy5.5^TDCNfs*** group. These results indicated that the doped ***Tel*** in ***TDCNfs*** could actively recognize the overexpressed AT1R under ischemic conditions, thus enhancing the MI-targeting effect.

### Studies on cardiac function post-MI

**Figure 7:**
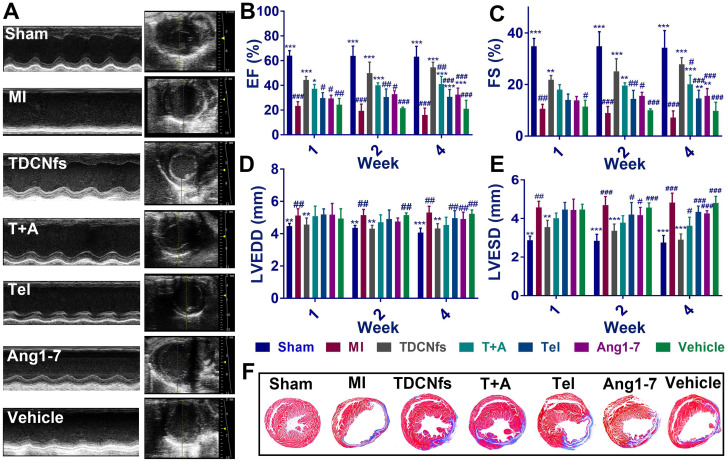
A) Representative echocardiograms (left) and measurements in different groups (4 weeks after LAD ligation); B-E) The percentage of left ventricular ejection fractions (LVEFs) , left ventricular fractional shortening (LVFS) and the values of left ventricular end-diastolic diameter (LVEDD), left ventricular end-systolic diameter (LVESD) evaluated by echocardiograms at 1,2,4 weeks after treatment; F) Representative Masson's trichome staining of different groups after MI. (Blue represents the area of MI. MI area = fibrotic area/left ventricular).^*^ *p* < 0.05 *vs.* MI, ^**^ p < 0.01 *vs.* MI, ^***^ *p* < 0.001 *vs.* MI, ^#^* p* < 0.05 *vs. **TDCNfs***, ^##^ *p* < 0.01 *vs. **TDCNfs***,^ ###^ *p* < 0.001 *vs. **TDCNfs***, n = 5.

The MI mouse model was established as described previously 58 and the mice were randomized into seven groups (***Sham***, ***MI***, ***TDCNfs***,*** T+A***, ***Tel***, ***Ang1-7***,*** vehicle***). Next, the different drugs were administered by tail vein injection every 2 days, while an equal volume of PBS was used for the Sham and MI groups (dosage in Table S2). Echocardiography was performed to evaluate the left ventricular (LV) function of different groups at 1, 2, and 4 weeks. As shown in Figure 7A, the mice from surgical groups underwent enlargement in the LV cavity and deteriorative heart function compared to those in the ***Sham*** group. Measurement of the left ventricular ejection fractions (LVEFs) at different time points revealed a progressive deterioration of cardiac function in the MI group, in which LVEFs were reduced to approximately 16% (at the 4th week), whereas those from the ***TDCNfs, T+A***, ***Tel***, ***Ang1-7*** groups indicated the maintenance of contractile function in the infarcted heart after the same time period with LVEFs values(at the 4th week) of 52.43%, 38.78%, 29.54%, and 31.84%, respectively, which demonstrated that ***TDCNfs***provided the highest cardiac protection (Figure 7B). Moreover, in comparison with the MI group, similar trends of improvement in other functional indicators were also observed, including fractional shortening (FS) values, left ventricular end-diastolic diameter (LVEDD) values, and left ventricular end-systolic diameter (LVESD) values (Figure 7C-E). These results indicated that ***TDCNfs*** treatment could significantly improve cardiac systolic and diastolic functions and prevent the deterioration of heart post-MI. Thereafter, Masson's trichrome staining was performed for histological observation of the sacrificed model mice to quantitatively evaluate the infarct size in the injured heart. Consistent with the results obtained in the echocardiography examination, the infarct size was reduced most significantly upon ***TDCNfs*** treatment in comparison to that observed in the MI group (15.88% *vs.* 36.60%). This value in other groups was 20.80% (***T+A***), 25.80% (***Tel***), 24.72% (***Ang1-7***), and 32.92% (***vehicle***); therefore, it was confirmed that ***TDCNfs*** offered the greatest protection against cardiac fibrosis (Figure 7F & Figure S28). Moreover, hematoxylin-eosin (H&E) staining of major organs and the serum index of renal function (creatinine, Cre) and liver function (alanine transaminase, ALT) suggested that the drugs rarely exhibited biotoxicity *in vivo* (Figure S29-31).

### Underlying mechanisms of therapeutic benefits determined *in vivo*

**Figure 8:**
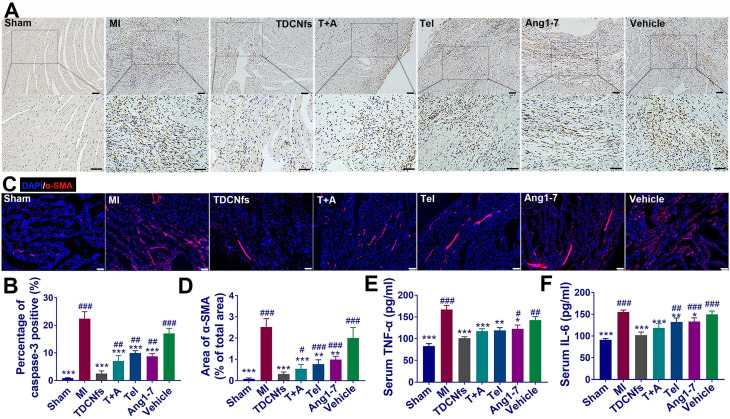
A) Immunohistochemical analysis of caspase-3 in the zone of bordering infarction region, blue represented normal nucleus and brown-yellow represented apoptosis-positive nucleus, scale bar = 50 µm; B) Quantitative analysis of caspase-3-positive cardiomyocytes in A; C) Immunofluorescence staining of α-SMA in the non-infarcted regions of different groups, scale bar = 75 µm; D) Quantification of α-SMA based on immunofluorescence area in C; E-F) Serum levels of pro-inflammatory cytokines IL-6 and TNF-α in different groups. ^*^p < 0.05 *vs.* MI, ^**^p < 0.01 *vs.* MI, ^***^p < 0.001 *vs.* MI, ^#^*p* < 0.05 *vs. **TDCNfs***, ^##^*p* < 0.01 *vs. **TDCNfs*****,** ^###^*p* < 0.001 *vs. **TDCNfs***, n = 5.

Considering the anti-apoptotic, anti-inflammatory, and anti-fibrotic effects exerted by ***TDCNfs**** in vitro*, we further validated the mechanisms underlying the amelioration of deteriorating heart function post-MI* in vivo*. The anti-apoptotic effect of ***TDCNfs*** was quantified using caspase-3 in the histological sections of the infarcted area. As shown in Figure 8A, abundant caspase-3 (stained in brown-yellow) expression was observed in the MI group, indicating that multiple cardiomyocytes underwent apoptosis under hypoxic-ischemic conditions. Caspase-3 expression in the ***Ang1-7***and ***Tel*** groups was reduced moderately owing to the distinct counter-regulatory effect of each agent on RAS. In contrast, caspase 3 expression was reduced drastically in the ***TDCNfs*** group compared to that in the other groups, which suggested the potent anti-apoptotic property of ***TDCNfs*** owing to the targeting and combinational effects on the blockade of RAS (Figure 8B).

Well-healed MI regions contain significant levels of extracellular matrix (ECM), while concurrent collagen deposition in the non-infarcted remote region results in adverse ventricular remodeling and further leads to cardiac failure 59,60. Immunofluorescence studies of α-SMA in the non-infarcted area were performed at 4 weeks post-treatment to observe fibrosis progression. Based on the quantified analysis, α-SMA was expressed abundantly in the MI group, whereas its expression was inhibited to a certain extent in the ***T+A***, ***Tel***, ***Ang1-7***, and ***vehicle*** groups. Notably, ***TDCNfs***treatment inhibited α-SMA expression more significantly than other drugs, which indicated that ***TDCNfs*** inhibited cardiac fibrosis in non-infarcted remote areas and prevented adverse ventricular remodeling more efficiently (Figure 8C). Further analysis of related fibrosis markers was performed, such as type I collagen and TGF-β1 (Figure S32). The results also demonstrated that ***TDCNfs***treatment could inhibit type I collagen and TGF-β1 expression more significantly than other drugs. Additionally, the levels of pro-inflammatory cytokines (TNF-α and IL-6) in the serum showed highly consistent trends of variation in different groups, which implied that ***TDCNfs*** exerted a better anti-inflammatory effect than other agents. Consequently, these results confirmed that the enhanced anti-apoptotic, anti-inflammatory, and anti-fibrotic effects of ***TDCNfs*** attributed to the synergistic counter-regulation of RAS could also enhance its therapeutic benefits *in vivo*.

## Conclusion

Currently, various drugs have been widely deployed to inhibit RAS, including ACEI, ARBs, and AT2 receptor agonists. However, the limited efficacy of monotherapy has put forward the urgent requirements for the development of combination therapy with amplified effectiveness 61,62. Thus, the exploration of innovative combination strategies for enhancing the clinical benefits of blocking RAS has garnered significant interest in the areas of laboratory research and drug discovery. Furthermore, few studies have explored the role of AT1R as a target in myocardial infarction, which endows ARB drugs with the ability to target therapy. In this study, we successfully developed ***Tel***-doped supramolecular nanofibers (***TDCNfs***) based on dual ligands for constructing a targeting and synergistic counter-regulatory RAS. ***TDCNfs*** simultaneously exhibited a moderate affinity toward MasR (attributed to ***Ang1-7***) and specific targeting of cardiomyocytes that overexpressed AT1R in the ischemic-hypoxic environment (predominantly attributed to ***Tel***). Taking advantage of the desirable targeting ability and the synergistic dual ligand-receptor interaction effects on mediating the downstream pathways, ***TDCNfs*** exerted enhanced protective action on damaged cardiomyocytes. Consequently, reduced apoptosis, alleviated inflammatory response, enhanced anti-fibrosis potential, and limited toxicity were observed, indicating the superior potential of ***TDCNfs*** in counteracting adverse cardiac outcome events post-MI. However, the susceptibility to proteolysis of self-assembled peptides remains a huge challenge, and their long-term systemic safety should be confirmed comprehensively. Hybridization with polymeric materials may be plausible for tracking down possible solutions 63,64. Overall, we believe that our strategy of combining bioactive peptides and small-molecule drugs through supramolecular self-assembly provides a feasible approach for efficient counter-regulatory RAS and the prevention of heart failure post-MI.

## Methods

### Materials

2-Cl-trityl chloride resin (loading: 0.939 mmol/g), fmoc-amino acids and o-benzotriazol-1-yl-N, N, N', N'-tetramethyluronium hexafluorophosphate (HBTU) were bought from GL Biochem (Shanghai). 4-Chloro-7-nitrobenzol-2-oxa-1, 3-diazole (NBD-Cl) and telmisartan were purchased from Sigma-Aldrich (USA). Cy5.5 NHS ester was obtained from APExBIO (USA). Recombinant MAS1 Protein (2 µg) was obtained from Abnova (China). Fetal bovine serum (FBS), Dulbecco's modified Eagle's medium (DMEM), trypsin, and Penicillin-streptomycin were obtained from Thermo Fisher (USA). DHE fluorescence probe, Apoptosis Analysis Kit, DAPI, and Hoechst 33342 dye were purchased from Molecular Probes (USA). CCK-8 kit was purchased from Dojindo Co. Ltd (Japan). The primary antibodies of anti-AT1R, anti-α-SMA, anti-Bax, anti-Bcl-2, anti-Histone 3, anti-Collagen I, anti-TGF-β1 and anti-cTnT were obtained from Abcam (Britain). The antibodies of anti-AKT, anti-p-AKT, anti-P65, anti-Caspase3 were purchased from Cell Signaling Technology (USA). ELISA kit for IL-6, TNF-α, CRE, ALT, TGF-β1 were bought from MSKBIO (China). All other solvents and reagents were commercially available and used without further purification, unless noted otherwise.

### Peptide synthesis

All peptides were prepared by solid-phase peptide synthesis; the specific synthetic route has been described in our previous work 20. The products were purified using High Performance Liquid Chromatography (LUMTECH, Germany). The mass spectrum of compounds was characterized by the TSQ Series Mass spectrometer system (Thermo Fisher, USA).

### Preparation of TDCNfs/^Cy5.5^TDCNfs

First, 10 mg of ***Tel*** was dissolved in 1 mL of anhydrous dimethyl sulfoxide and stirred for 10 min at 40 °C.*** SAA1-7***/***^Cy5.5^SAA1-7***was dissolved in phosphate-buffered saline (pH 7.4) at a concentration of 10 mg/mL. Next, 400 µL of ***SAA1-7***/***^Cy5.5^SAA1-7***was brought to boil and then cooled to 40 °C; different volumes of ***Tel*** solution (molar ration control) at 40 °C and PBS were then added to the ***SAA1-7***/***^Cy5.5^SAA1-7***solution for the final volume to 500 µL. The reaction mixtures were incubated at 40 °C for 2 h. Unassembled*** Tel*** was removed by centrifugation at 15000 × g for 15 min. Finally, the supernatant was discarded; ***TDCNfs***/^***Cy5.5***^***TDCNfs***was formed after lyophilization and was ready for use after dilution in PBS (pH 7.4) at 5 mg/mL.

### Characteristics of compounds

Time-dependent (0, 30, 60, 120 min) samples in the process of ***SAA1-7*** doped with*** Tel***, and the samples of ***TDCNfs*** colloidal solution (10 mg/mL) for 1 h, 1 month, and 2 months were prepared and observed using TEM (JEM-2100F, Japan). ***TDCNfs***(diluted in PBS, 10 mg/mL) were prepared in a gradient molar ratio (15%, 30%, 50%) of ***Tel*** and the fluorescence emission spectra (BIO-RAD, λexc = 260 nm) were recorded. Different concentrations of ***TDCNfs*** and*** SAA1-7***were prepared to determine CMC using dynamic light scattering (BI-200SM, Brookhaven, USA). Infrared Spectroscopy of ***TDCNfs, SAA1-7*** was performed in IR-Prestige 21 FTIR Spectrophotometer (Shimadzu, Japan). The rheology test of ***SAA1-7***, ***TDCNfs***, ***^Cy5.5^ TDCNfs*** was done on an AR 1500ex (TA Instrument) system. The details are given in Supporting Information.

### Surface plasmon resonance spectroscopy

Recombinant Human MAS1 protein (Abnova, H00004142-G01) was immobilized on the surface of a CM5 sensor chip, and ***TDCNfs***, ***SAA1-7***, and ***Ang1-7***, were diluted in PBS at concentrations ranging from 0.001 to 10 μM for affinity measurement and kinetic tests (PlexArray HT).

### Cell isolation and culture

The isolation of primary neonatal rat cardiomyocytes (NRCMs) and neonatal rat cardiac fibroblasts (NRCFs) were conducted as previously reported 65. Oxygen/glucose deprivation was facilitated to simulate hypoxia during MI. In brief, NRCMs cultured in PBS were placed in a humidified environment at 37 °C in a tri-gas incubator equilibrated with 1% O_2_, 5% CO_2_, and 94% N_2_ for 1 h, after which the drugs were added at the appropriate concentrations, and the cells were incubate under the same conditions for 2-3 h.

### Cytotoxicity and Cardioprotective Effects of *TDCNfs*

Cytotoxicity was quantified by measuring the viability of NRCMs and NRCFs treated with serial concentrations of ***TDCNfs*** (0.01-100 μΜ, diluted in growth medium, 89% DMEM + 10% FBS + 1% PS) for 24 and 48 h under normoxia. The cardioprotective effect was quantified by measuring the viability of NRCMs treated with gradient concentrations of ***TDCNfs*** (0.01-100 μΜ) or 10 μΜ of ***TDCNfs, Tel***+***Ang1-7***,*** Tel***,*** Ang1-7***, and ***Vehicle***under OGD conditions. Cell viability was measured in CCK-8 assays according to the manufacturer's instructions. The absorbance was measured spectrophotometrically using a microplate reader (Bio-Rad Benchmark Plus) at 450 nm.

### *In vitro* immunofluorescence assay

For studying ***TDCNfs*** co-localization, NF-κB distribution in NRCMs, and the extent of NRCF differentiation, the cells were washed with PBS and fixed with 4% paraformaldehyde for 20 min after intervention. Next, the cells were blocked by incubating with goat serum for 30 min and incubated with primary antibodies overnight at 4 °C. The primary antibodies used here included anti-AT1R (1:200 dilution), anti-cTNT (1:500 dilution), anti-P65 (1:800 dilution), and anti‐α-SMA (1:400 dilution). The cells were incubated with FITC-labeled Goat Anti-Rabbit IgG (1:200 dilution) or Cy3-labeled Goat Anti-Mouse IgG (1:200 dilution) for 2 h at room temperature. The cells were counterstained with DAPI and imaged using a fluorescence microscope (Leica Dmi8, Germany).

### Cellular uptake *in vitro*

NRCMs were cultured in 6-well plates at 1 × 10^6^ cells per well under normoxic or OGD conditions for 1 h. Next, the cells were treated with ***TDCNfs***(50 μM), ***SAA1-7*** (50 μM), and ***^NBD^Ang1-7***(50 μM) and cultured for 2 h, harvested, and centrifuged. Subsequently, the cells were washed and resuspended in PBS. Cellular uptake was evaluated using Flow Cytometer (BD FACS Verse, USA).

### Intracellular TUNEL and ROS measurements

The anti-apoptotic and anti-ROS effects of ***TDCNfs*** in NRCMs were observed by TUNEL staining using a one-step TUNEL Apoptosis Assay Kit or dihydroethidium (DHE) according to the manufacturer's protocol. The cells were counterstained with DAPI and were observed under a fluorescence microscope (details in Supporting Information).

### Western blot analysis

AKT, p-AKT, Bax, Bcl-2, cleaved caspase-3, P65, TGF-β1, Histone3, AT1R, GAPDH were quantified by western blot analysis. Protein concentration was determined using the bicinchoninic acid (BCA) method. SDS-PAGE was conducted to separate the proteins in each sample (30-50 µg of protein per lane), which were subsequently transferred to a PVDF membrane. The membranes were treated with blocking buffer for 2 h at room temperature and then incubated overnight with primary antibodies at 4 °C. The primary antibodies used included anti-Phospho-AKT (1:1000 dilution), anti-AKT (1:1000 dilution), anti-caspase-3 (1:1000 dilution), anti-Bcl-2 (1:100 dilution), anti-Bax (1:1000 dilution), anti-P65 (1:800 dilution), anti-TGF-β1 (1:1000 dilution), anti-Histone 3 (1:1000 dilution) and anti-AGTR1 (1:1000 dilution). The results were normalized by comparison to parallel western blots for glyceraldehyde 3-phosphate dehydrogenase (GAPDH, 1:8000 dilution). Optical density was measured using an image processing analysis program.

### Quantitative real-Time Polymerase Chain Reaction (RT-qPCR)

Total RNA was extracted from the cultured cells using TRIzol (Invitrogen, USA). mRNA was reverse-transcribed using the PrimeScript RT reagent kit (TaKaRa, Japan) according to the manufacturer's instructions. RT-qPCR was performed using SYBR Premix (Takara, Japan) in a Step One Plus real-time PCR system (Applied Biosystems, USA). The RT-qPCR primers used in this study are listed in Table S3. The value was normalized to that of *GAPDH*, and relative gene expression was determined using the ^ΔΔ^Ct method.

### Conditioned Medium (CM) interference experiments

CM from untreated or compound-treated cells were produced as follows. NRCMs were seeded in 35 mm culture dishes at 1 × 10^6^ cells per well and incubated in growth medium containing different compounds or in media devoid of compounds for 12 h. Next, the media were replaced with serum-free medium, and the cells were cultured under hypoxia for 6 h. The CM was collected and centrifuged to culture NRCFs after a 12 h starvation period. Immunofluorescence of α-SMA was analyzed as per protocol after 24 h.

### Scratch test of NRCFs

The NRCFs were treated with medium containing different compounds and 100 nM Ang II after serum-free incubation for 12 h, and subsequently, a scratch was generated in the cell monolayer using a 1 mL tip. The migration of the cells was monitored by imaging at 0 and 24 h. The area between the scratch was measured at each time point and the migration index was calculated as follows:


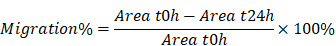


### Mouse MI modeling and grouping

Adult male C57BL/6 mice (20-30 g, 8-10 week) were purchased from the laboratory animal center of Southern Medical University, China. The protocols were approved according to the Zhujiang Hospital of Southern Medical University animal care and use committee guidelines, which conform to the guide for the care and use of laboratory animals published by the US National Institutes of Health (8th edition, 2011). For the MI model, detailed methods were seen in Supporting Information. The mice that underwent LAD ligation treatment were randomly divided into MI,*** TDCNfs***,*** Tel+ Ang1-7***, ***Tel***, ***Ang1-7***, and ***vehicle*** groups. Next, 100 µL of different drugs were administered intravenously through tail vein injection every 2 days for 3 weeks, while the mice in the Sham and MI groups were treated with an equal volume of PBS.

### Live animal imaging experiments

***^Cy5.5^TDCNfs***, ***^Cy5.5^SAA1-7***, and ***^Cy5.5^Ang1-7*** were injected intravenously into the mice via tail vein at a dose of 0.2 mg. The images were captured at the prefixed time. Next, the mice were sacrificed to harvest their hearts and major organs. The samples were imaged at an excitation wavelength of 630 nm and an emission wavelength of 700 nm using an *in vivo* imaging system (FX PRO, BRUKER, Germany). The images were analyzed using the BRUKER Molecular Imaging Software. The targeting ability of compounds was defined by HTI = heart fluorescence emission/liver fluorescence emission.

### *In vivo* fluorescence assay

Frozen sections were prepared *in vivo*, as described previously 64. For drugs distribution, near-infrared laser imager (GE Amersham Typhoon, Sweden) was used to capture full cross section of hearts. For co-localization experiments, the mice hearts were dissected 24 h post-injection of drugs. Anti-AT1R primary antibody (1:200 dilution) was used as the primary antibody. For fibrosis investigation, the frozen sections were incubated with primary antibodies against α-SMA (1:400 dilution). The staining signals were visualized using FITC-labeled Goat Anti-Rabbit IgG (1:200 dilution) or Cy3-labeled Goat Anti-Rabbit IgG (1:200 dilution). The sections were counterstained with DAPI and examined under a laser confocal scanning microscope (Leica TCS SP8, Germany).

### Echocardiography

Twenty-eight days after surgery, the mice were anesthetized through the inhalation of isoflurane (1-1.5%) in O_2_ and echocardiographic examination was performed using a Vevo 2100 System equipped with a 30 MHz transducer (FUJIFILM Visual Sonics, Canada). The LVEDD, LVESD, LVEFs, and LVFS were measured. All measurements were repeated for at least three consecutive pulsation cycles and the data were averaged for statistical analysis.

### Histological analysis

The mice were sacrificed at 4 weeks after the induction of MI, and hearts and major organs were harvested and fixed in 4% paraformaldehyde overnight and embedded in paraffin. The hearts were sectioned to 5 µm along the short axis transversely across the infarct zone. The major organs were sectioned to 5 µm along the short axis. Following deparaffinization and dehydration, the samples underwent H&E and Masson's trichrome staining. The sections were observed under a microscope to evaluate the therapeutic effect and safety of the drugs. The collagen and LV areas were measured using the Image-Pro Plus software (version 6.0; Media Cybernetics, Silver Spring, MD, USA). Infarct size (%) was calculated as the ratio of the collagen area to the LV area.

### Immunohistochemical analysis

The heart tissues were fixed in 4% paraformaldehyde, dehydrated in ethanol, cleared in xylene, and embedded in paraffin. The samples were then cut into 6 μm sections on a microtome and deparaffinized. The sections were blocked by incubating with 1% BSA at room temperature for 30 min and then incubated overnight with anti-cleaved caspase-3 (1:700 dilution), anti-AT1R (1:100 dilution), anti-Collagen I (1:200 dilution) at 4 °C. Next, the sections were washed and incubated with secondary antibodies (1:500 dilution) at room temperature. The tissue sections were incubated with diaminobenzidine and counterstained with hematoxylin, dehydrated, and mounted before viewing under a microscope.

### Enzyme-linked Immunosorbent Assay (ELISA)

The serum and CM were collected at the indicated time points from the different groups of mice. The levels of TNF-α, IL-6, ALT, Cre, and TGF-β1 were measured using ELISA kits according to the manufacturer's protocol. The absorbance was measured at 450 nm using a microplate reader.

### Statistical analysis

All statistical analyses were performed using the SPSS statistical software (IBM SPSS Statistics version 25.0, SPSS Inc., Chicago, IL, USA). The values were expressed in terms of mean ± standard deviation. A one-way ANOVA test of multiple comparisons followed by Dunnett's post-hoc test was used in all analyses. *P* < 0.05 was considered statistically significant.

## Supplementary Material

Supplementary figures and tables are available online free of charge online, including details of synthesis and characterization, specific procedures of experiments *in vitro* and *in vivo*.

## Abbreviations

MI: myocardial infarction
RAS: renin-angiotensin system
ACE: angiotensin-converting enzyme
ACEI: angiotensin-converting enzyme inhibitors
Ang II: angiotensin II
AT1R: angiotensin II type 1 receptor
ARB: angiotensin receptor blocker
ACE 2: angiotensin-converting enzyme 2
Ang1-7: angiotensin 1-7
MasR: Mas receptor
Tel: telmisartan
SAA1-7: self-assembly angiotensin 1-7
TDCNfs: telmisartan doped co-assembly nanofibers
OGD: oxygen/glucose deprivation
NRCMs: neonatal rat cardiomyocytes
NRCFs: neonatal rat cardiac fibroblasts
LAD: left anterior descending coronary artery
HTI: heart-targeting index
LVEFs: left ventricular ejection fractions
LVFS: left ventricular fractional shortening
LVEDD: left ventricular end-diastolic diameter
LVESD: left ventricular end-systolic diameter
