# Supplementary material for: Dual-ligand supramolecular nanofibers inspired by the renin-angiotensin system for the targeting and synergistic therapy of myocardial infarction
Source: Theranostics. 2021 Jan 27;11(8):3725–41. doi: 10.7150/thno.53644 (PMC7914367; doi:10.7150/thno.53644)
Supplement: Supplementary file 1 — Supplementary figures and tables are available online free of charge online, including details of synthesis and characterization, specific procedures of experiments in vitro and in vivo. [file thnov11p3725s1.pdf]

Dual-ligand supramolecular nanofibers inspired by the renin-angiotensin system for the targeting and synergistic therapy of myocardial infarction

Zhanpeng Wen,[1#] Jie Zhan,[2#] Hekai Li,[1] Guanghui Xu,[3] Shaodan Ma,[1] Jianwu Zhang,[4] Zehua Li,[1] Caiwen Ou,[1] Zhimou Yang,[3,5,6]* Yanbin Cai,[1]* and Minsheng Chen[1]*

[1]Guangdong Provincial Biomedical Engineering Technology Research Center for Cardiovascular Disease, Department of Cardiology and Laboratory of Heart Center, Sino-Japanese Cooperation Platform for Translational Research in Heart Failure, Zhujiang Hospital, Southern Medical University, Guangzhou 510280, China.

[2]Shunde Hospital, Southern Medical University, the First People's Hospital of Shunde, Foshan 528300, China.

[3]School of Pharmaceutical Sciences, Southern Medical University, Guangzhou 510515, China.

[4]Department of Cardiology, Nanfang Hospital, Southern Medical University; Guangzhou 510515, China.

[5]Key Laboratory of Bioactive Materials, Ministry of Education, State Key Laboratory of Medicinal Chemical Biology, College of Life Sciences, Nankai University, Tianjin 300071, China.

[6]Jiangsu Center for the Collaboration and Innovation of Cancer Biotherapy, Cancer Institute, Xuzhou Medical University, Xuzhou 221004, Jiangsu, China.

[#] These authors contributed equally to this work

***Corresponding authors:** Zhimou Yang, E-mail: yangzm@nankai.edu.cn; Yanbin Cai, E-mail: skyer1@smu.edu.cn; Minsheng Chen, E-mail: gzminsheng@vip.163.com.

Contents

S1 Synthesis and characterization

S1.1 Synthesis of NBD-β-Alanine

S1.2 Synthesis of peptides

S1.3 Drug Loading and Encapsulation Efficiency

S1.4 Transmission electron microscopy (TEM)

S1.5 Critical micelle concentration (CMC)

S1.6 Fluorescence emission spectra

S1.7 Infrared Spectroscopy

S1.8 Rheology test

S2 Cell experiments

S2.1 Cells isolation

S2.2 Cell viability detection

S2.3 *In vitro* co-localization

S2.4 Intracellular TUNEL and ROS measurements

S3 Animal experiment

S3.1 Mouse MI modeling

S3.2 Live animal imaging experiments

S3.3 *In vivo* co-localization

S4 Supporting figures and tables

S1 Synthesis and characterization

S1.1 Synthesis of NBD-β-Alanine and peptides:

980 mg of Alanine (1.1 equiv.), 4.14 g of K_2CO_3 (3 equiv.), and 2 g of NBD-Cl (1 equiv., 10 mmol) were added in 15 mL H_2O. 20 mL of MeOH was then added dropwise under N_2 protect (low yields if without nitrogen protection) and stirred for further 3 h at room temperature (LC-MS detection). After that, MeOH was removed by a rotary evaporator. The obtained solution was acidified to pH 3 to obtain precipitate (NBD-β-Alanine, yield: 87%).

S1. 2 Synthesis of peptides

NBD-^{D}F^{D}F^{D}Y^{D}E^{D}EG-DRVYIHP (***SAA1-7***), NBD-G-DRVYIHP (^{NBD}***Ang1-7***), NBD-^{D}F^{D}F^{D}Y^{D}E^{D}EG-YRVIPHD (***Vehicle***), NH_2-^{D}F^{D}F^{D}Y^{D}E^{D}EG-DRVYIHP-COOH，NH_2-G-DRVYIHP-COOH were prepared by standard solid-phase peptide synthesis using 2-chlorotrityl chloride resin. The first Fmoc-protected amino acid was loaded onto the resin at the C-terminal. The Fmoc group of the amino acid was deprotected with 20% piperidine in anhydrous N, N'-dimethylformamide. The next Fmoc-protected amino acid was activated by N, N-diisopropylethylamine and coupled to the free amino group using O-(benzotriazole)-N, N, N′, N′-tetramethyluronium hexafluorophosphate (HBTU) as the coupling reagent. The growth of the peptide chain was allowed to proceed according to the standard Fmoc solid-phase peptide synthesis protocol. After the last coupling step, the peptide derivatives were cleaved from the resin with a mixture of 1% trifluoroacetic acid and 99% dichloromethane for 10 minutes, then dried via rotary evaporation followed by diethylether precipitation.

Cy5.5-^{D}F^{D}F^{D}Y^{D}E^{D}EG-DRVYIHP ($^{Cy5.5}$***SAA1-7***) and Cy5.5-G-DRVYIHP ($^{Cy5.5}$***Ang1-7***) were prepared through liquid-phase synthesis with the ratio of Cy5.5-NHS easter and peptides at 1: 1.5, respectively. Briefly, the peptides were dissolved in DMSO and adjusted pH to 8-9, then Cy5.5-NHS Ester was added to solution and stirred at room temperature for 24 h in the dark.

The products were purified by High Performance Liquid Chromatography (HPLC) and characterized by mass spectrometry.

S1.3 Drug Loading and Encapsulation Efficiency

Tel (dissolved in DMSO) was mixed with ***SAA1-7*** (dissolved in PBS) (pH 7.4, 10 mg/mL) at molar ratios of 15%, 30%, 50%, and 70% (n = 3). After preparation, the mixtures were analyzed using LC-MS (LCMS-20AD, Shimadzu) after lyophilization. DL and EE were calculated using the following equation.

$$\text{DL\%}= \frac{W\ recovered\ drug}{W\ recovered\ drug + W\ recovered\ peptide} * 100\%$$

$$\text{EE\%}=\frac{W\ recovered\ drug}{W\ added\ drug} * 100\%$$

In vitro **Drug Release of *Tel* and The Stability of *TDCNfs*.** ***TDCNfs*** (25 mg) was dissolved in PBS (5 mL) and the pH was adjusted to 7.4. Proteinase K was added at a concentration of 0.138 mg/mL and the reaction mixtures were incubated at 37 °C for 24 h. Next, 500 μL of the sample was removed at each time point and the release of ***Tel*** was analyzed, while the remained ***SAA1-7*** in the sample was analyzed using LC-MS simultaneously.

S1.4 Transmission electron microscopy

The ***SAA1-7*** with 50% molar ratio of ***Tel*** was incubated at 40 °C for 0 min, 30 min, 60 min, and 120 min, respectively. TEM samples in different time points were prepared. Besides, the samples of ***TDCNfs*** diluted in PBS (pH=7.4) for 1 h, 1 month, 2 months were prepared. The sample preparation procedures: 10 μL samples of each were placed on a carbon-coated copper grid and incubated for 30 seconds to allow the peptide nanostructures to adhere to the substrate, then rinsed twice with ultrapure water. The samples were then stained with a saturated uranyl acetate solution and placed in a desiccator overnight before analysis.

S1.5 Critical micelle concentration (CMC)

TDCNfs and ***SAA1-7*** were dissolved in MilliQ water with gradient concentrations. The CMC values were determined by dynamic light scattering (DLS) and the light scattering intensity was recorded for each concentration. The lower CMC values

representative better assembly capacity.

S1.6 Fluorescence emission spectra

SAA1-7 solution/hydrogel (diluted in PBS, 10 mg/mL), ***Tel*** (diluted in DMSO,10 mg/mL) and ***TDCNfs*** (diluted in PBS, 10 mg/mL) contanining gradient molar ratio (15%, 30%, 50%) of ***Tel*** were prepared to measured fluorescence emission spectra (BIO-RAD, λexc = 265 nm).

S1.7 Fourier-transform Infrared Spectroscopy

Fourier-transform infrared spectroscopy of the samples was performed in IR-Prestige 21 FTIR Spectrophotometer (Shimadzu, Japan). FT-IR spectra were recorded for ***TDCNfs***, ***SAA1-7***. The measurement range was 4000–750 cm^{-1}, scan number for per sample was 16, and resolution was 4 cm^{-1}.

S1.8 Rheology

The rheology test was done on an AR 1500 ex (TA Instrument) system; 25 mm parallel plate at a gap of 500 μm was used during the experiment. The hydrogel of ***SAA1-7***, ***TDCNfs***, $^{CY5.5}$***TDCNfs*** were characterized by the mode of dynamic frequency sweep in the region of 0.1–100 rad/s at a strain of 1%.

S2 Cell experiments

S2.1 Cells isolation

Neonatal Sprague-Dawley (SD) rats (1–3 d old, weighing 5-7 g, means 6.1 ± 0.7 g) were obtained from the Experimental Animal Centre of Southern Medical University. Primary neonatal rat cardiomyocytes (NRCMs) and neonatal rat cardiac fibroblasts (NRCFs) were isolated from the hearts of neonatal SD rats.

Briefly, after the digestion of hearts, the cells were collected and suspended in DMEM medium supplemented with 10% FBS and incubated with 95% O_2, 5% CO_2. After 2 h, attached cardiac fibroblasts (NRCFs) continued to culture in fresh DMEM medium supplemented with 10% FBS. Unattached cells were plated for another 2 h to remove non-myocytes to obtain NRCMs and further cultured in cardiomyocyte growth medium (89% DMEM+10% FBS+1% PS) for 96 h before use.

S2.2 Cell viability

To detect the effect of different treatments on cardiomyocytes and cardiac

fibroblasts viability *in vitro*, we used serial concentrations of ***TDCNfs*** (0.01 μM, 0.1 μM, 1 μM, 10 μM, 100 μM) diluted in growth medium to incubate with NRCMs and NRCFs for 24 h and 48 h under normoxia condition. To determine the effect of ***TDCNfs*** on NRCMs under OGD condition, NRCMs cultured in PBS were placed in a humidified environment at 37 °C in a tri-gas incubator equilibrated with 1% O_2, 5% CO_2 and 94% N_2 for 1 h, after that medium were replaced by ***TDCNfs*** (diluted in PBS), the cells were then incubated under the same conditions for 2-3 h. To compare the effect of different compounds on cardiomyocytes viability under OGD condition, NRCMs were cultured in PBS for 1 h under 1% O_2 and further incabuted with 10 μM of ***TDCNfs, T+A***, ***Tel***, ***Ang1-7***, ***Vehicle*** for 2-3 h, respectively. The cell viability was measured by CCK-8 assays. After different treatments, the medium was removed and replaced with 100 μL of DMEM medium containing 10 μL of CCK-8 kit solution according to the manufacturer's protocol. The absorbance was measured by a microplate reader at wavelengths of 450 nm.

S2.3 *In vitro* co-localization

After being cultured in OGD condition for 1 h, NRCMs were cultured with ***TDCNfs*** (50 μM), ***SAA1-7+Tel*** (50 μM) and ^{NBD}***Ang1-7+Tel*** (50 μM) for further 2 h. PBS was then used to wash cells for three times and 4% paraformaldehyde was added to fix cells for 20 min at room temperature. After being blocked with goat serum for 30 min, rabbit anti-AT_1R primary antibody (1:200 dilution) was added to incubate with NRCMs overnight at 4 °C. After being washed with PBS for three times, Cy3-labeled Goat Anti-Rabbit IgG (1:200 solution) was added and incubated with cells for 2 h at room temperature. Cells were then stained with DAPI. The fluorescence images were obtained by the fluorescence microscope (λexc =532 nm for AT_1R, λexc = 488 nm for NBD, λexc = 405 nm for DAPI).

S2.4 Intracellular TUNEL and ROS Measurements

NRCMs were incubated in OGD condition for 1 h, and then cultured with PBS or different compounds (diluted in PBS) at 10 μM under the same conditions for 2-3 h. The cells were then fixed in 4% paraformaldehyde and apoptotic cells were dyed by TUNEL staining using a one-step TUNEL apoptosis assay kit according to the

manufacturer's protocol. Cells were then stained with DAPI. The TUNEL positive cells were observed by the fluorescence microscope (λexc = 488 nm for TUNEL, λexc = 405 nm for DAPI).

Intracellular ROS was quantified with dihydroethidium (DHE). After incubated with different treatments under OGD condition, NRCMs were then cultured with DMEM medium containing 10 mM of DHE for 30 min at 37 °C and then washed twice with PBS. Nucleuses of NRCMs were dyed by Hoechst 33342 stain solution for 30 min at 37 °C. Intracellular ROS were detected by the fluorescence microscope (λexc = 532 nm for DHE, λexc = 405 nm for Hoechst 33342).

S3 Animal experiment

S3.1 Mouse MI modeling

Adult male C57BL/6 mice (20-30g, 8-10 weeks old) were purchased from the laboratory animal center of Southern Medical University. For the MI model, mice were subjected to permanent left anterior descending (LAD) ligation as described previously [1]. In brief, the animals were anesthetized using 1.5% sodium pentobarbital (50 mg/kg) through intraperitoneal injection, and mice were ventilated with a rodent ventilator subsequently. We exposed mice hearts by the fourth intercostal space incision and a subsequent pericardium removal. The LAD was ligated using an 8-0-prolene suture permanently to perform MI generation. Sham group mice underwent the same surgical procedure without LAD ligation.

S3.2 Live animal imaging experiments

To investigate their biodistribution, $^{Cy5.5}$***TDCNfs*** was intravenously injected into the mice with or without myocardial infarction at a dose of 0.2 mg and $^{Cy5.5}$***SAA1-7***, $^{Cy5.5}$***Ang1-7*** was intravenously injected into the mice with myocardial infarction at the same dose. At 1 h, 12 h and 24 h post-injection, the images were captured. After 24 h, mice were sacrificed, hearts and the major organs were excised. Images were captured at an excitation wavelength of 630 nm and an emission wavelength of 700 nm using an *in vivo* imaging system. Images were analyzed using the BRUKER Molecular Imaging Software.

S3.3 *In vivo* co-localization

The hearts of mice were then dissected after intravenous injection of compounds at 24 h. Immunofluorescence staining on frozen section of heart samples as reported previously [2]. Anti-AT_1R primary antibody (1:200 dilution) was used as the primary antibodies. Staining signals were visualized with FITC-labeled Goat anti-Rabbit IgG (1:200 dilution). The sections were counterstained with DAPI and examined using a laser confocal scanning microscopy.

S4. Supporting Figures and Tables

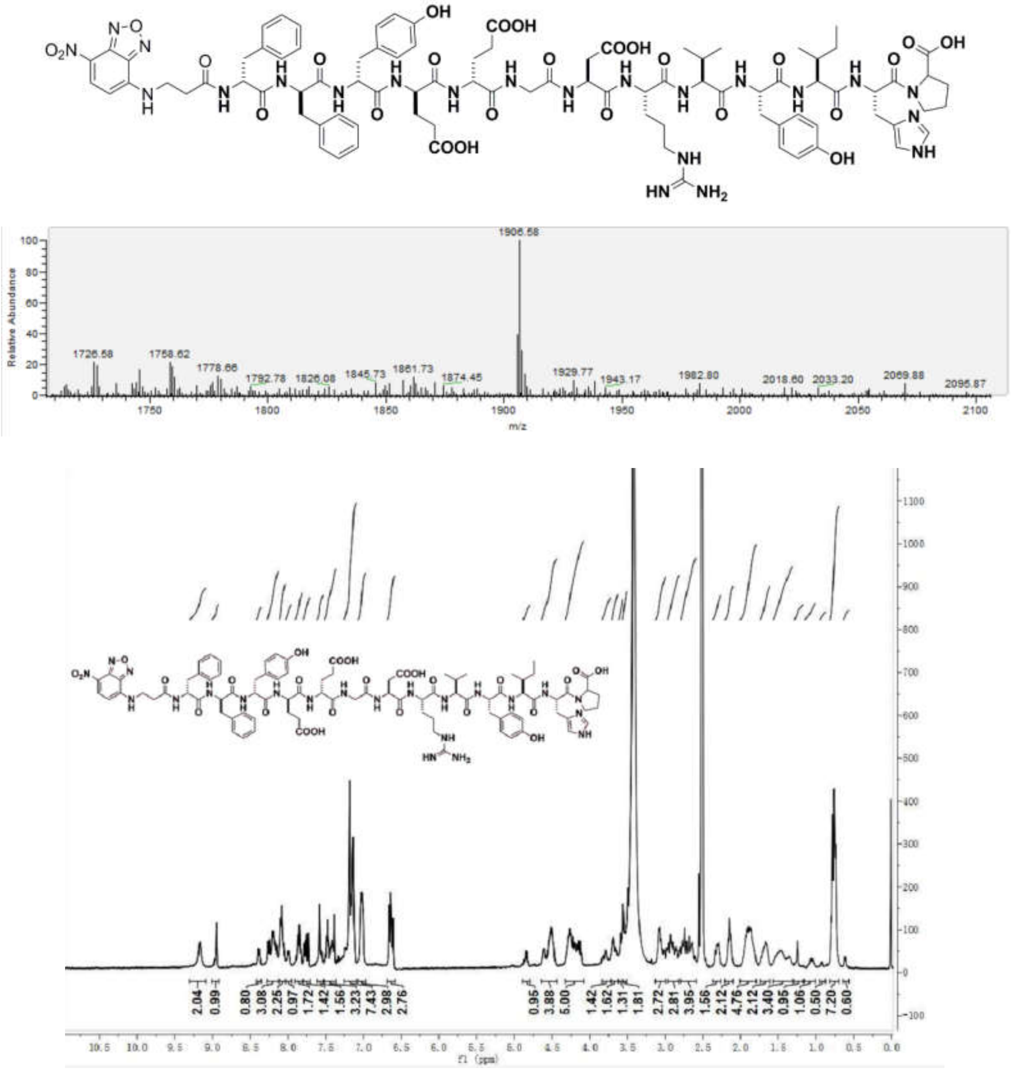

Figure S1. Chemical structure and characterization of NBD- ^{D}F^{D}F^{D}Y^{D}E^{D}EG-DRVYIHP (***SAA1-7***), Calc. M = 1906.58, obsvd. M = 1906.58.

^{1}H NMR (400 MHz, DMSO) δ 9.17 (s, 2H), 8.95 (s, 1H), 8.39 (d, J = 7.4 Hz, 1H), 8.27 – 8.13 (m, 3H), 8.09 (d, J = 7.9 Hz, 2H), 7.99 (d, J = 8.1 Hz, 1H), 7.86 (dd, J = 9.1, 8.8 Hz, 2H), 7.76 (dd, J = 9.7, 8.0 Hz, 1H), 7.57 (d, J = 10.0 Hz, 2H), 7.52 – 7.36 (m, 3H), 7.25 – 7.09 (m, 7H), 7.02 (dd, J = 7.9, 3.9 Hz, 3H), 6.63 (dd, J = 9.4, 8.4 Hz, 3H), 4.84 (d, J = 7.4 Hz, 1H), 4.64 – 4.44 (m, 4H), 4.18 (ddd, J = 9.1, 17.7, 11.5 Hz, 5H), 3.84 –

3.73 (m, 1H), 3.67 (d, J = 9.7 Hz, 2H), 3.58 (d, J = 9.6 Hz, 1H), 3.54 (d, J = 9.5 Hz, 2H), 3.05 (dd, J = 9.6, 9.7 Hz, 3H), 2.96 – 2.82 (m, 3H), 2.69 (dt, J = 9.0, 14.1 Hz, 4H), 2.31 (d, J = 9.4 Hz, 2H), 2.21 – 2.10 (m, 2H), 2.01 – 1.79 (m, 5H), 1.67 (s, 2H), 1.41 (d, J = 9.1 Hz, 3H), 1.25 (s, 1H), 1.06 (d, J = 7.4 Hz, 1H), 0.93 (s, 1H), 0.77 (dd, J = 9.9, 7.1 Hz, 7H), 0.61 (d, J = 6.4 Hz, 1H).

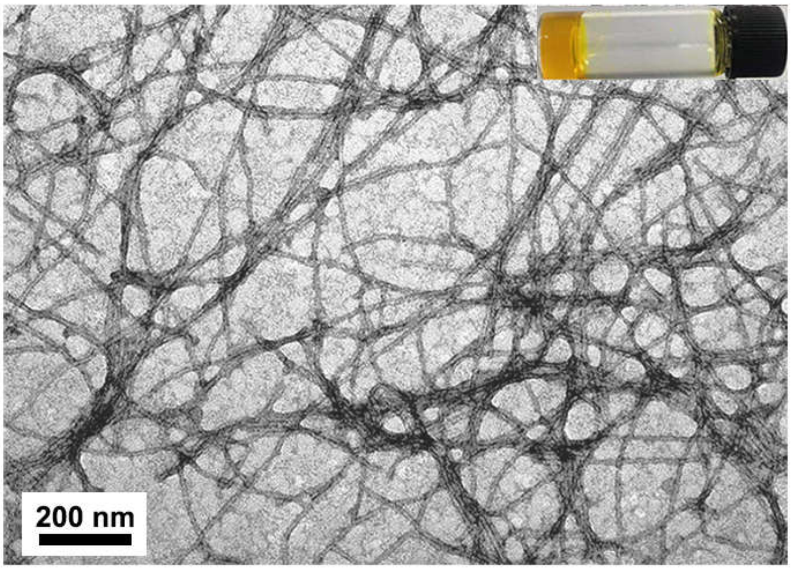

Figure S2. TEM image of NBD-^{D}F^{D}F^{D}Y^{D}E^{D}EG-DRVYIHP (***SAA1-7***), the image inserted represented the final hydrogel formed at a concentration of 1.0 wt%.

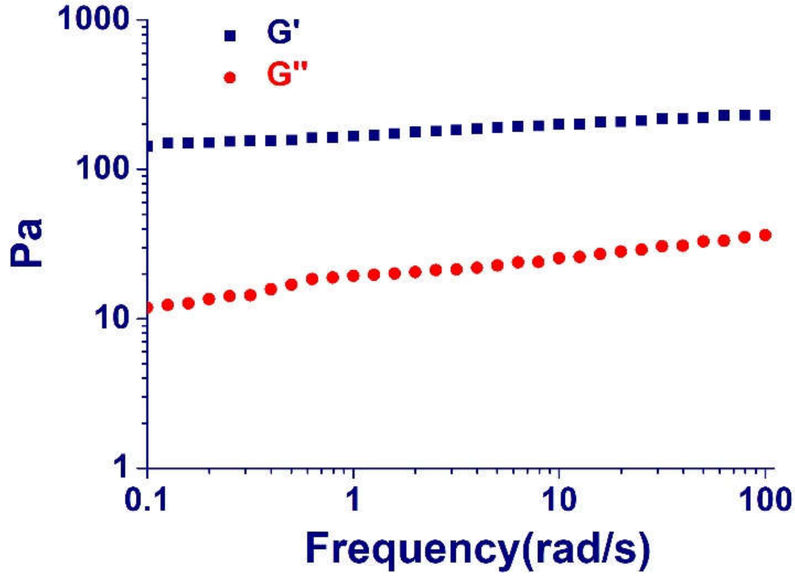

Figure S3. Rheology tests of ***SAA1-7***

.

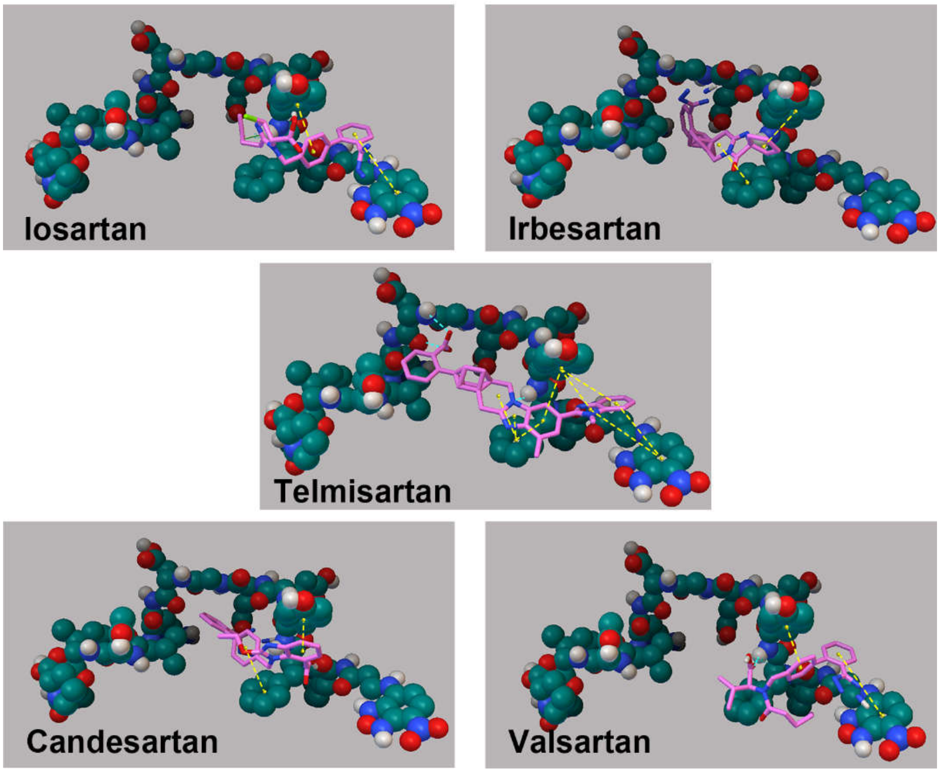

Figure S4. Chemical structures with interactions between ***SAA1-7*** and ARBs. The yellow imaginary lines represent π–π stacking interactions, the cyan imaginary lines represent H-bond interactions.

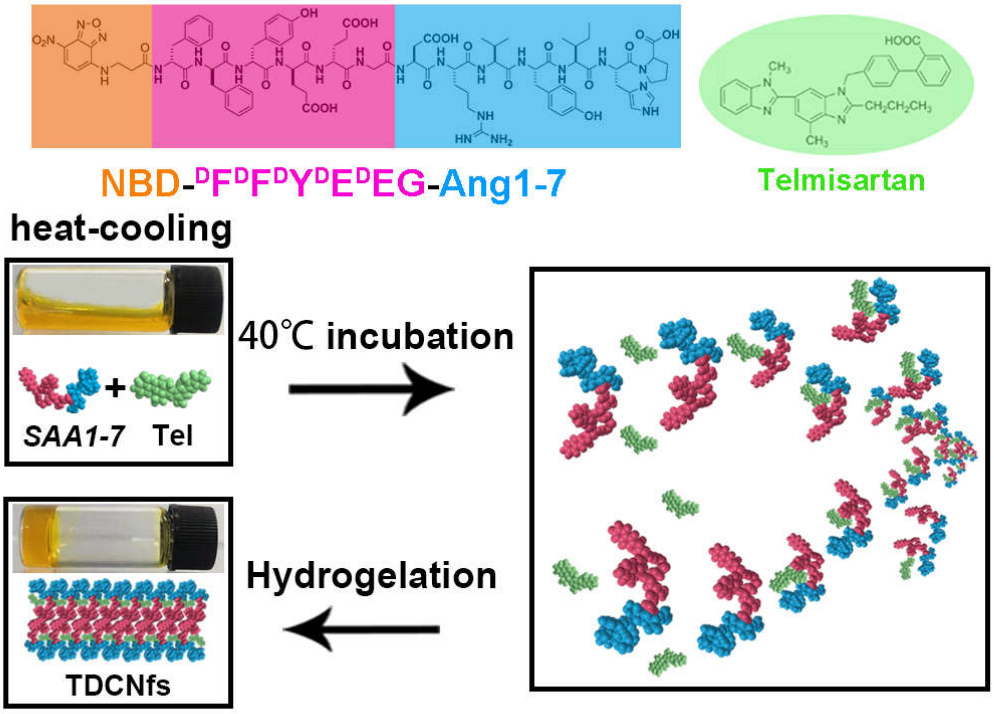

Figure S5. Scheme of co-assembly process of ***SAA1-7*** and ***Tel.***

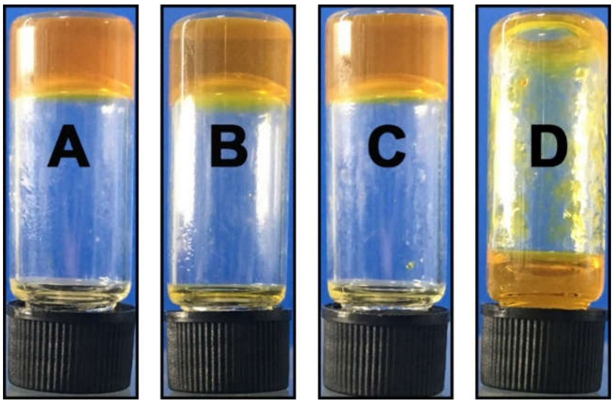

Figure S6. Different molar ratio of ***Tel*** doped in ***SAA1-7*** (A=15%, B=30%, C=50%, D=70%).

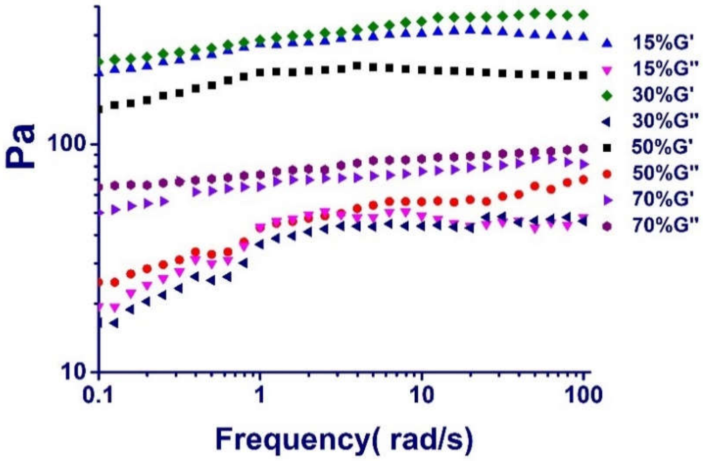

Figure S7. Rheology tests of ***TDCNfs*** with various molar concentrations of ***Telmisartan***

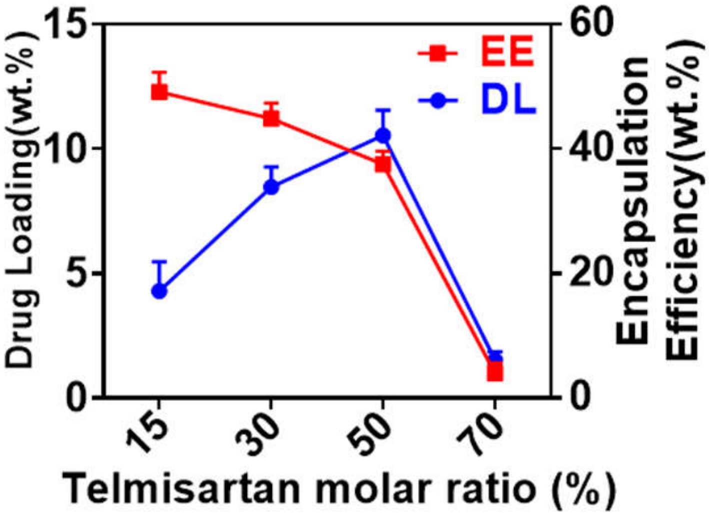

Figure S8. Drug loading and encapsulation efficiency of ***TDCNfs*** with different molar ratio of ***Tel***.

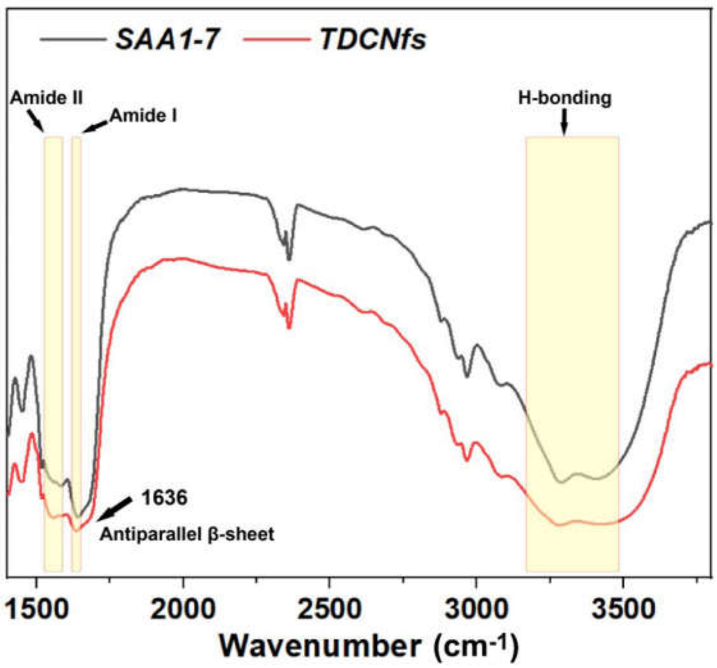

Figure S9. FT-IR of ***SAA1-7*** and ***TDCNfs***.

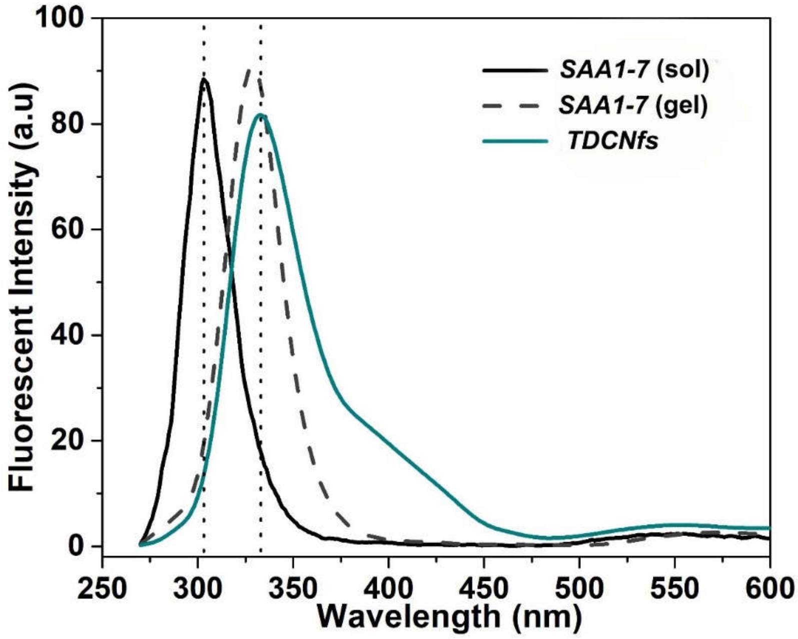

Figure S10. Fluorescence emission spectra of ***SAA1-7*** (sol/gel) and ***TDCNfs***.

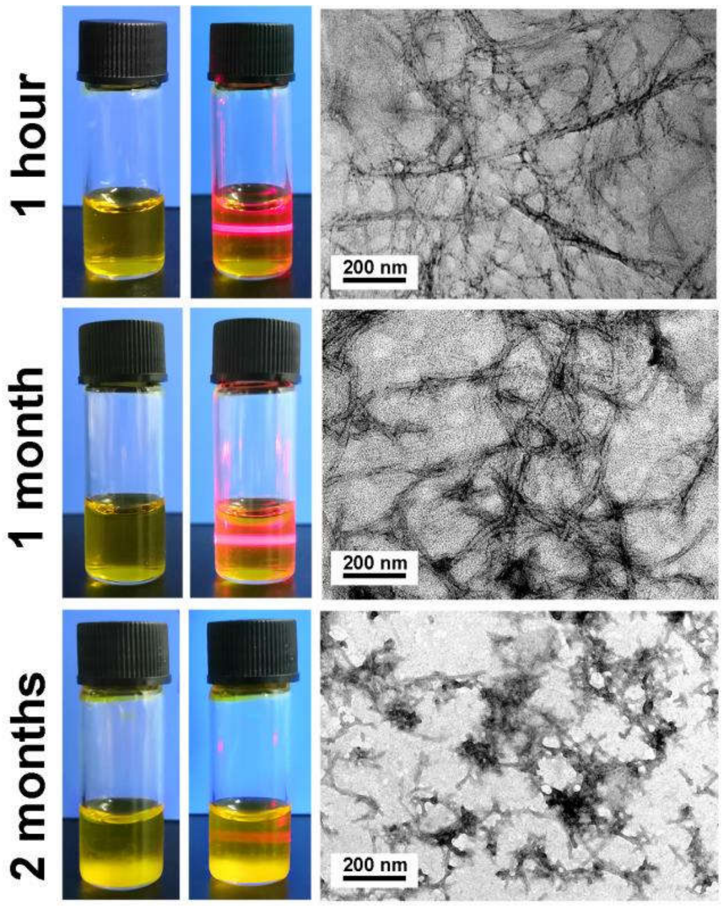

Figure S11. Optical and TEM images of ***TDCNfs*** alone with time.

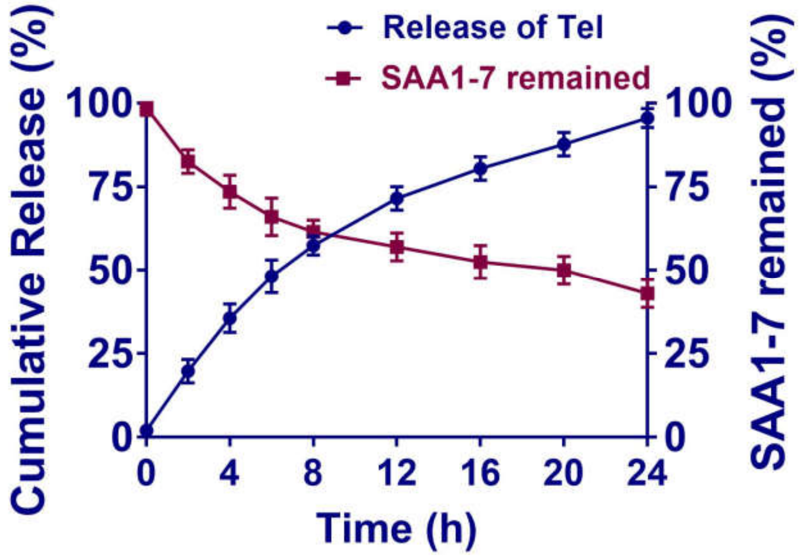

Figure S12. Release profile of ***Tel*** and the stability of ***TDCNfs*** (5 mg/mL) against proteinase K (0.138 mg/mL) digestion in PBS buffer solution (pH = 7.4).

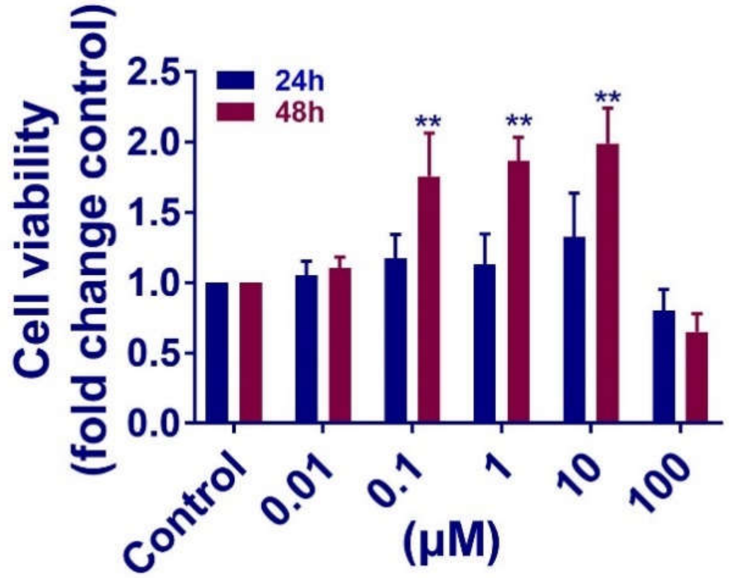

Figure S13. Biocompatibility of ***TDCNfs*** on NRCMs under normal conditions. Control group means without the treatment of ***TDCNfs***. (** p <0.01 *vs.* control group for 48 h; n = 6 for each group). All data were obtained from three independent experiments.

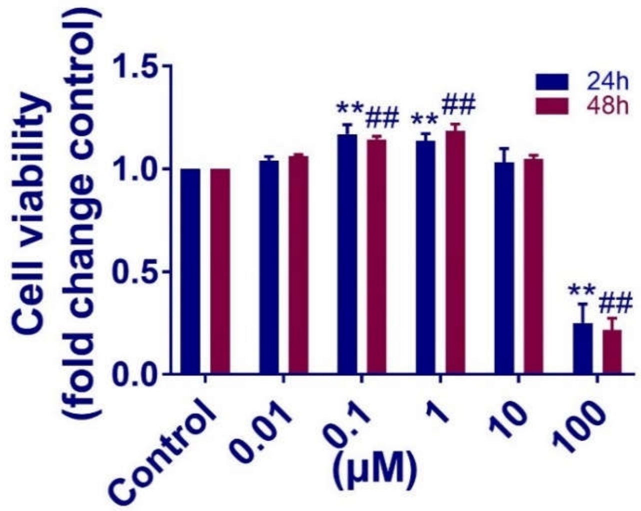

Figure S14. Biocompatibility of ***TDCNfs*** on NRCFs under normal conditions. Control group means without the treatment of ***TDCNfs***. (** p <0.01 *vs.* control group for 24 h; $^{\#\#}$ p < 0.01 *vs.* control group for 48 h; n = 6 for each group). All data were obtained from three independent experiments.

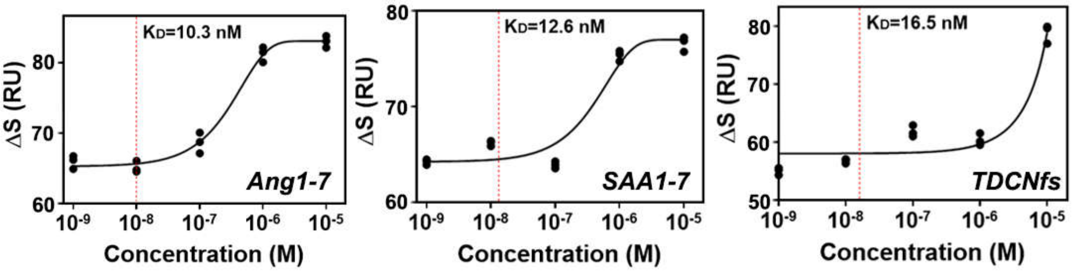

Figure S15. SPR response units of ***Ang1-7***, ***SAA1-7*** and ***TDCNfs*** with rhMasR at different concentrations and the fitting curves of the results. Their KD values are labeled in the corresponding curves.

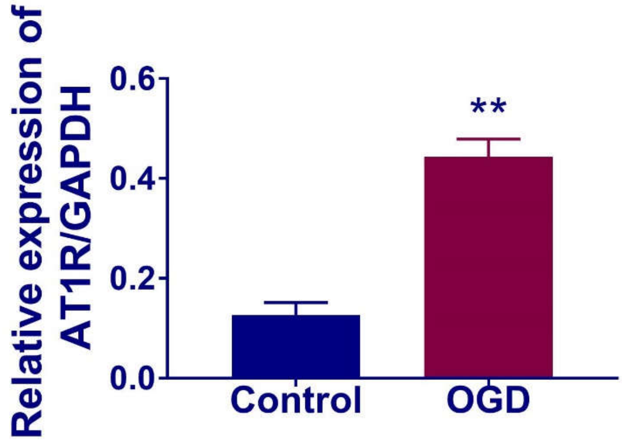

Figure S16. Quantification of AT1R using densitometry. $^{**}p < 0.01$ *vs.* Control, n = 3.

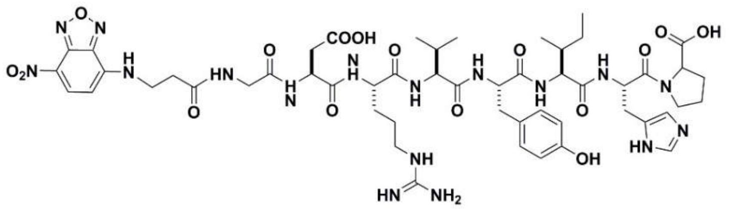

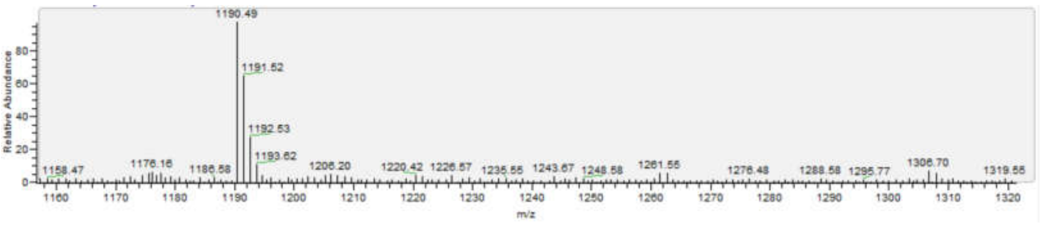

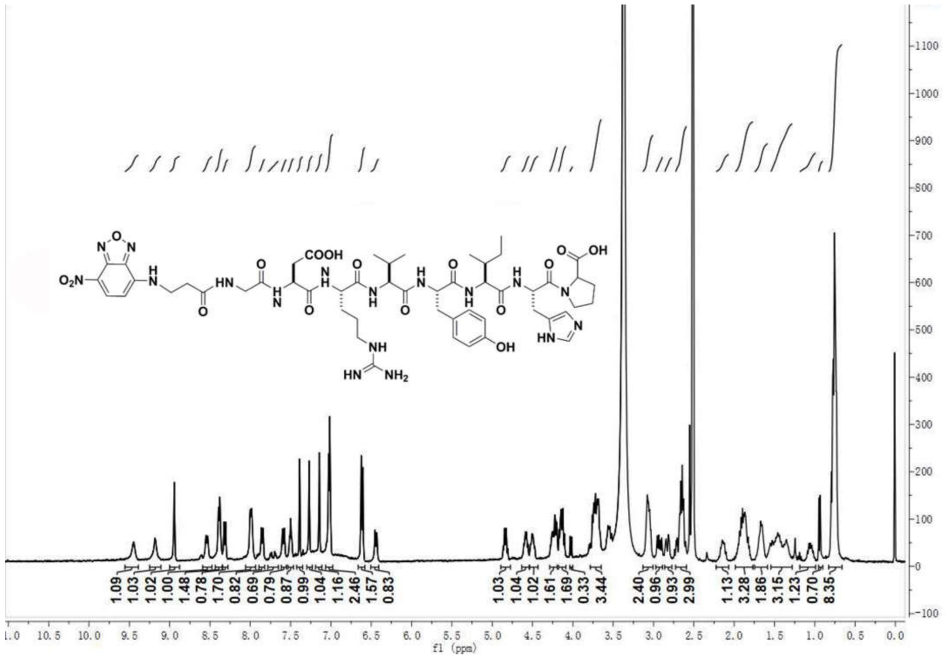

Figure S17. Chemical structure and characterization of NBD-G-DRVYIHP (^{NBD}***Ang1-7***), Calc. M =1190.49, obsvd. M = 1190.49.

^{1}H NMR (400 MHz, DMSO) δ 9.45 (s, 1H), 9.18 (s, 1H), 8.94 (s, 1H), 8.54 (d, J = 7.9 Hz, 1H), 8.39 (d, J = 7.4 Hz, 1H), 8.32 (d, J = 7.6 Hz, 1H), 7.99 (d, J = 6.3 Hz, 2H), 7.85 (d, J = 8.6 Hz, 1H), 7.78 – 7.65 (m, 1H), 7.59 (d, J = 9.2 Hz, 1H), 7.50 (s, 1H), 7.37 (d, J = 9.4 Hz, 1H), 7.27 (s, 1H), 7.14 (s, 1H), 7.02 (d, J = 5.9 Hz, 2H), 6.61 (d, J = 8.2 Hz, 2H), 6.44 (d, J = 8.8 Hz, 1H), 4.84 (dd, J = 9.6, 7.5 Hz, 1H), 4.63 – 4.54 (m, 1H), 4.50 (d, J = 4.2 Hz, 1H), 4.29 – 4.19 (m, 2H), 4.14 (dd, J = 9.2, 8.7 Hz, 2H), 4.02 (d, J = 6.6 Hz, 1H), 3.78 – 3.65 (m, 3H), 3.07 (s, 2H), 2.93 (dd, J = 9.8, 7.3 Hz, 1H), 2.83 (d, J = 9.7 Hz, 1H), 2.73 – 2.59 (m, 3H), 2.15 (s, 1H), 1.98 – 1.77 (m, 3H), 1.63 (d, J = 9.3 Hz, 2H), 1.54 – 1.28 (m, 3H), 1.19 – 0.99 (m, 1H), 0.94 (d, J = 6.7 Hz, 1H), 0.83 – 0.66 (m, 8H).

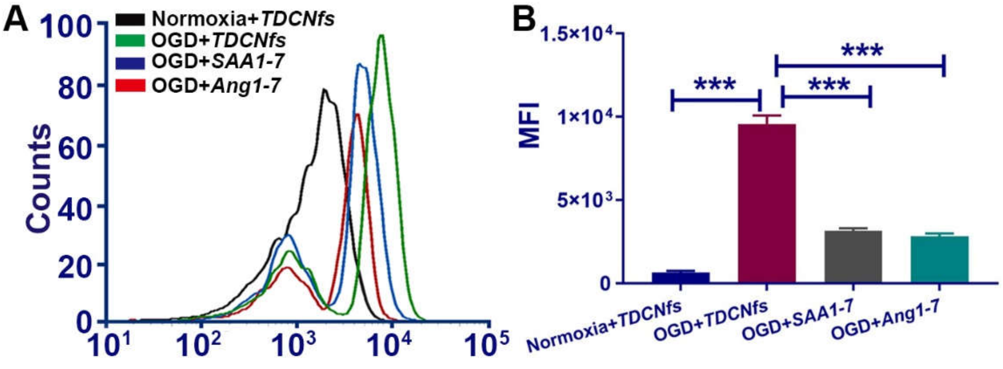

Figure S18. A) The intracellular fluorescence intensity and B) quantification analysis of NRCMs treated with ***TDCNfs***, ***SAA1-7***, ***Ang1-7*** under normoxic and OGD conditions, measured using flow cytometry. *** *p* <0.001.

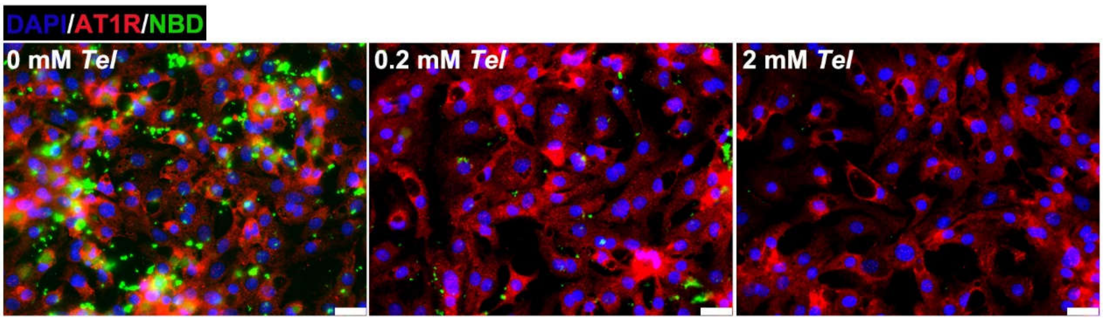

Figure S19. Co-localization of AT1R (red) with ***TDCNfs*** (green) in NRCMs under OGD conditions when pre-saturation with different concentration of free ***Tel***, scale bar = 25 μm.

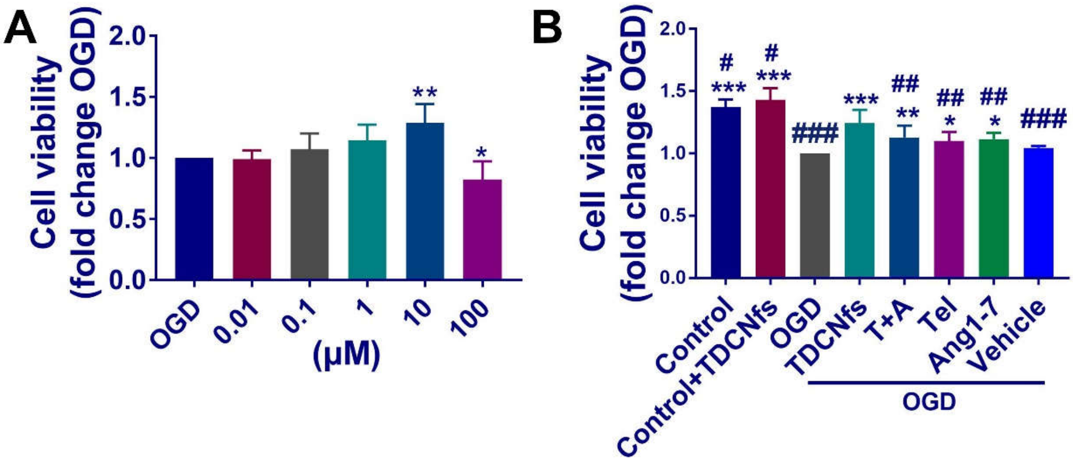

Figure S20. Cell viability of NRCMs cultured with A) gradient concentrations of TDCNfs and B) different group compounds under hypoxia conditions ($^{*}p < 0.05$ *vs.* OGD group, $^{**}p < 0.01$ *vs.* OGD group, $^{***}p < 0.001$ *vs.* OGD, $^{\#\#}p < 0.01$ *vs.* ***TDCNfs***, $^{\#\#}p< 0.01$ *vs.* ***TDCNfs***, $^{\#\#\#}p< 0.001$ *vs.* ***TDCNfs***; n = 6 for each group); All data were obtained from three independent experiments.

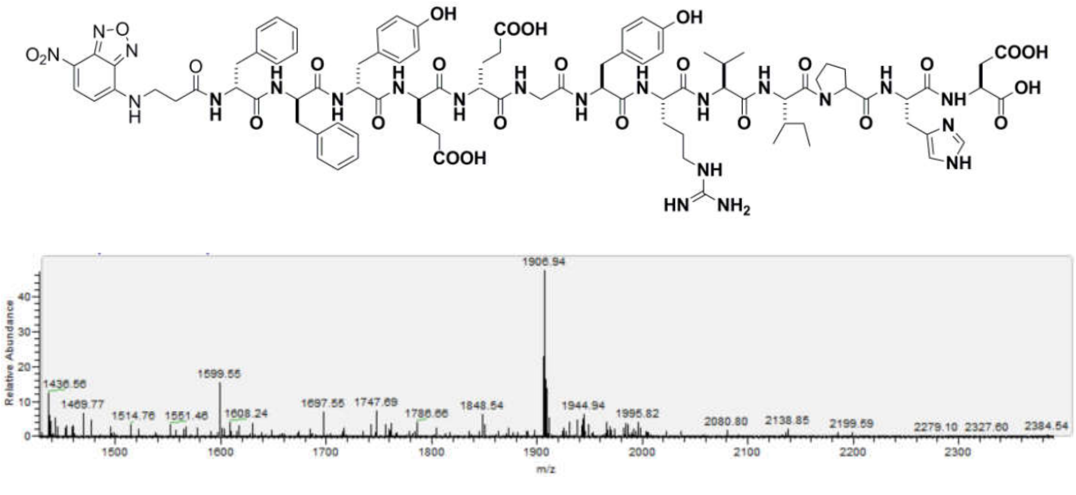

Figure S21. Chemical structure and characterization of NBD-^{D}F^{D}F^{D}Y^{D}E^{D}EG-YRVIPHD (***vehicle***), Calc. M =1906.94, obsvd. M= 1906.94

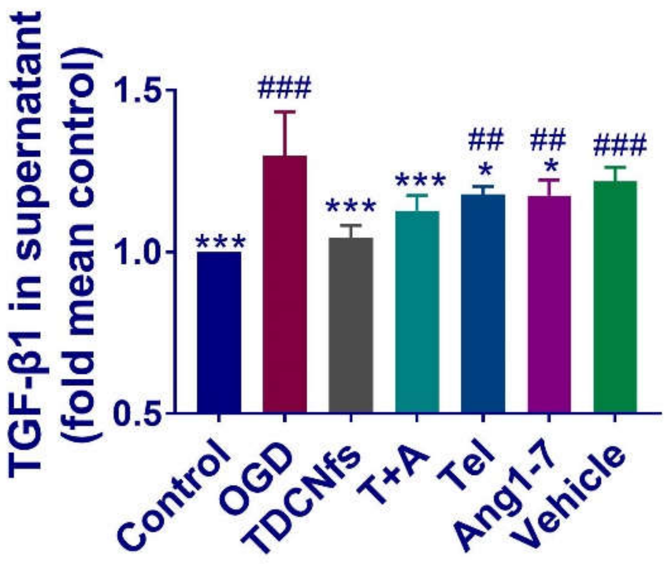

Figure S22. Expression of TGF-β1 in conditioned medium from hypoxia-induced cardiomyocytes pre-treated with different compounds (10μM). ($^{*}p < 0.05$ *vs.* OGD, *** $p < 0.001$ *vs.* OGD, $^{\#\#}$ $p < 0.01$ *vs.* ***TDCNfs***, $^{\#\#\#}$ $p < 0.001$ *vs.* ***TDCNfs***). All data were obtained from three independent experiments.

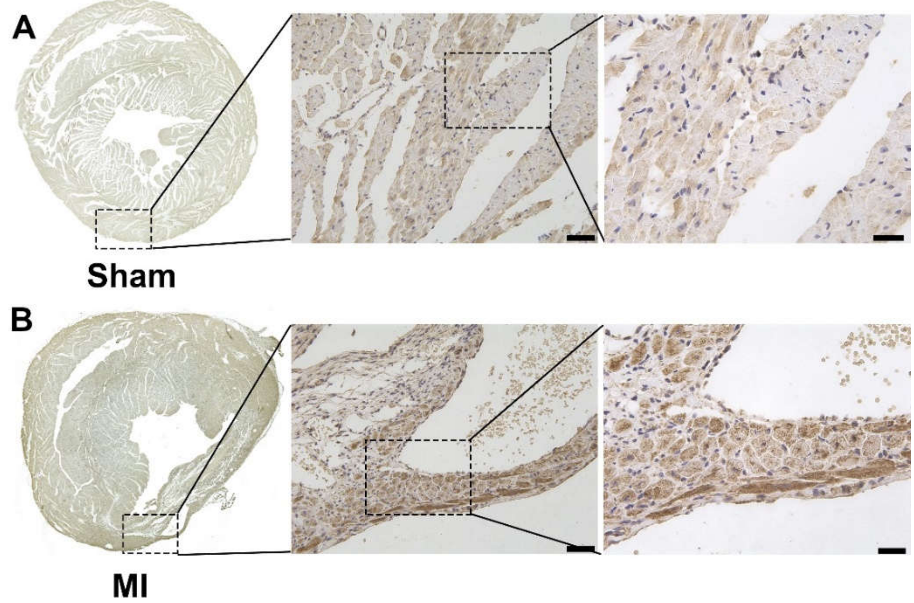

Figure S23. Representative images of immunohistochemistry for AT1R in the zone of infarction region, blue represented nucleus and brown-yellow represented AT1R-positive cells, scale bar = 50 μm.

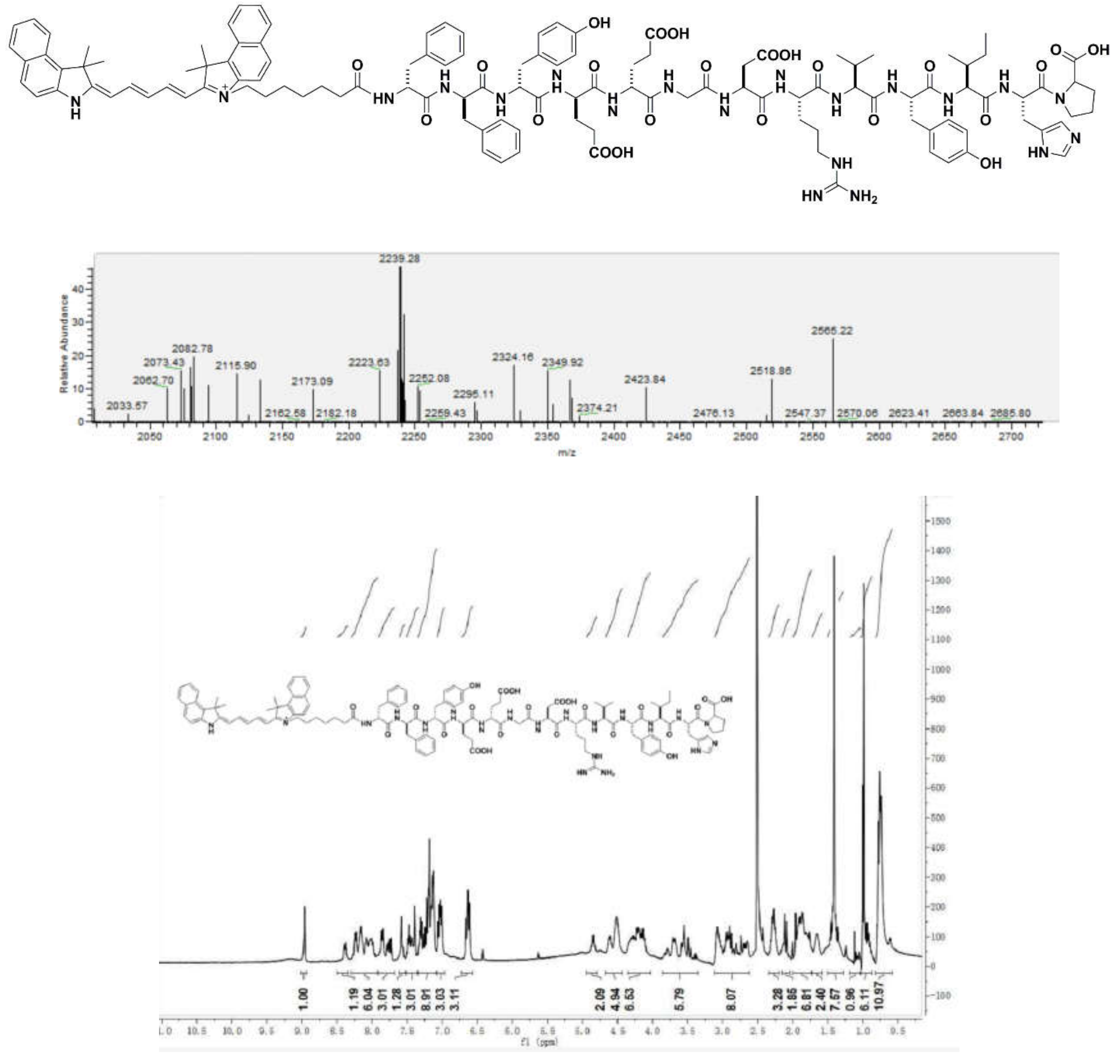

Figure S24. Chemical structure and characterization of Cy5.5-^{D}F^{D}F^{D}Y^{D}E^{D}EG-DRVYIHP ($^{Cy5.5}$***SAA1-7***), Calc. M =2237.61, obsvd. $[M+H]^+$ = 2239.28.

^{1}H NMR (400 MHz, DMSO) δ 8.96 (s, 1H), 8.39 (d, J = 7.7 Hz, 1H), 8.30 – 7.92 (m, 6H), 7.91 – 7.68 (m, 3H), 7.58 (s, 1H), 7.51 – 7.34 (m, 3H), 7.35 – 7.08 (m, 9H), 7.03 (dd, J = 9.2, 8.2 Hz, 3H), 6.73 – 6.57 (m, 3H), 4.85 (d, J = 7.2 Hz, 2H), 4.56 (d, J = 9.4 Hz, 5H), 4.35 – 4.03 (m, 7H), 3.86 – 3.35 (m, 6H), 3.12 – 2.62 (m, 8H), 2.34 – 2.19 (m, 3H), 2.10 (d, J = 9.4 Hz, 2H), 2.00 – 1.74 (m, 7H), 1.65 (s, 2H), 1.42 (s, J = 9.1 Hz, 8H), 1.09 (dd, J = 9.1, 10.0 Hz, 1H), 1.04 – 0.87 (m, 6H), 0.83 – 0.58 (m, 11H).

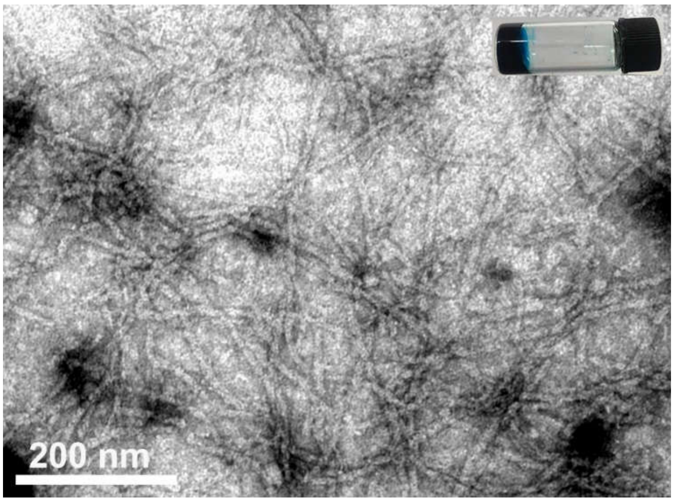

Figure S25. The TEM and optical (inserted graph) images of $^{Cy5.5}$***SAA1-7*** hydrogel at concentration of 1.0 wt%.

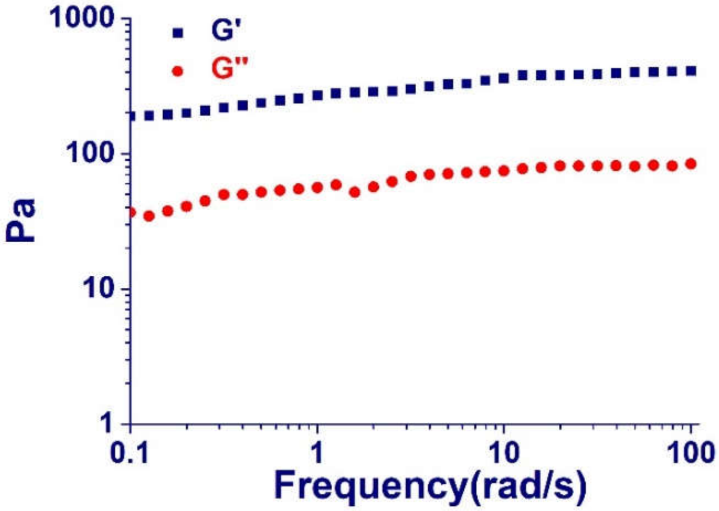

Figure S26. Rheology tests of $^{Cy5.5}$ ***TDCNfs***

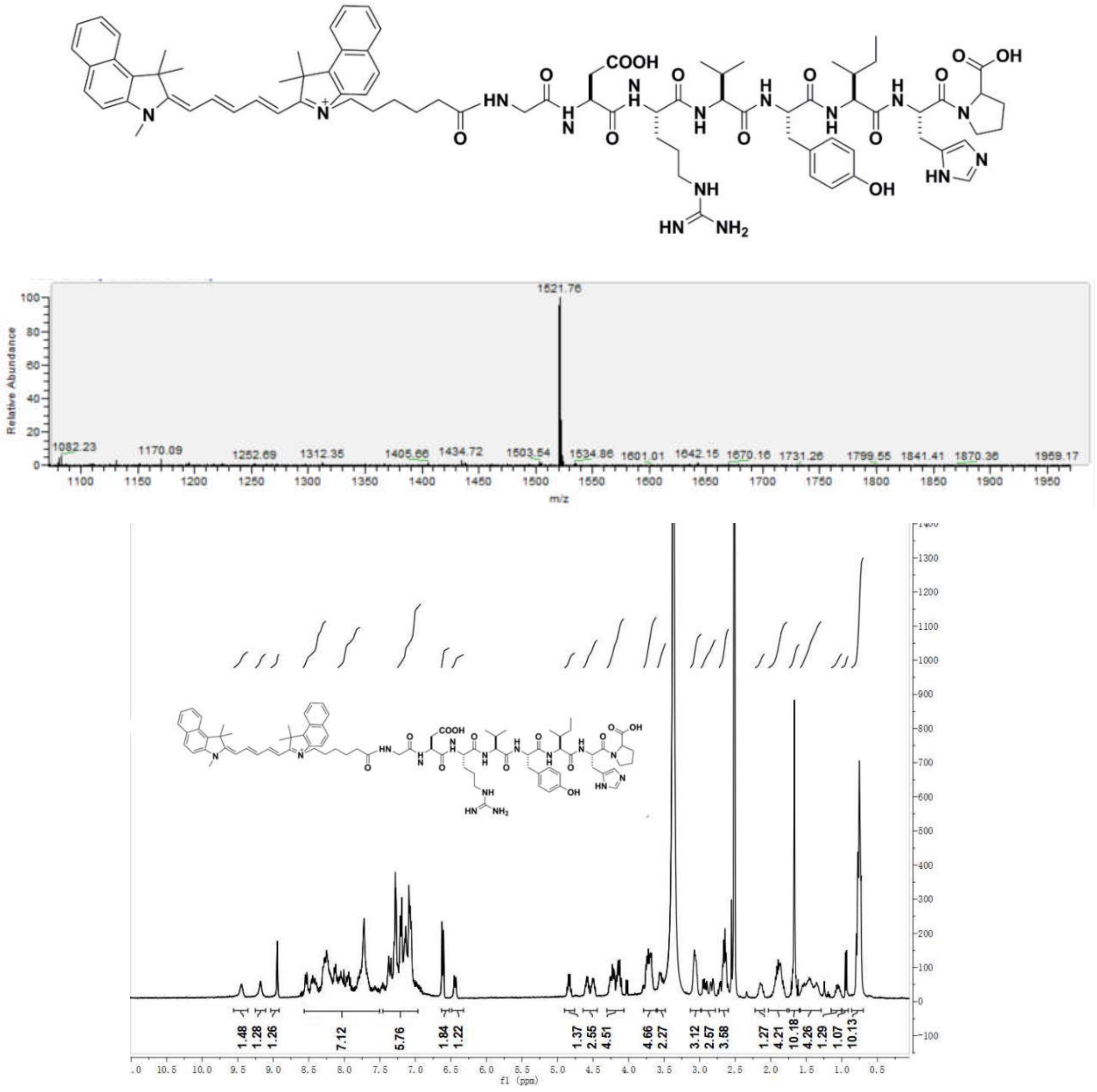

Figure S27. Chemical structure and characterization of Cy5.5-G-DRVYIHP ($^{Cy5.5}$***Ang1-7***), Calc. M =1521.76, obsvd. M =1521.76.

^{1}H NMR (400 MHz, DMSO) δ 9.45 (s, 1H), 9.18 (s, 1H), 8.94 (s, 1H), 8.58 – 7.56 (m, 7H), 7.42 – 6.92 (m, 6H), 6.61 (d, J = 8.2 Hz, 2H), 6.44 (d, J = 8.8 Hz, 1H), 4.84 (dd, J = 9.6, 7.5 Hz, 1H), 4.64 – 4.44 (m, 3H), 4.31 – 4.07 (m, 5H), 3.79 – 3.61 (m, 5H), 3.60 – 3.48 (m, 2H), 3.07 (s, 3H), 2.98 – 2.78 (m, 3H), 2.73 – 2.60 (m, 4H), 2.15 (s, 1H), 2.03 – 1.77 (m, 4H), 1.67 (s, 10H), 1.58 – 1.29 (m, 4H), 1.06 (dd, J = 9.4, 6.8 Hz, 1H), 0.94 (d, J = 6.7 Hz, 1H), 0.86 – 0.70 (m, 10H), 0.01 (s, 1H).

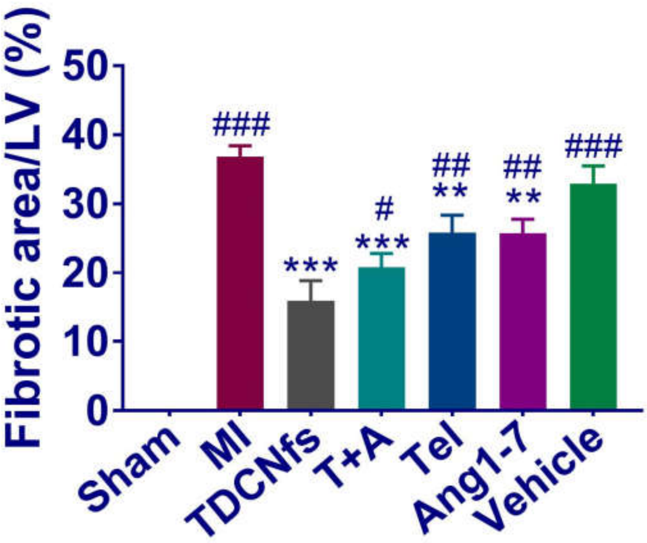

Figure S28. Myocardial fibrotic area of different groups (fibrotic area/left ventricular). $^{**}p < 0.01$ *vs.* MI, $^{***}\ p < 0.001$ *vs.* MI, $^{\#}\ p < 0.\ 05$ *vs.* ***TDCNfs***, $^{\#\#}\ p < 0.01$ *vs.* ***TDCNfs***, $^{\#\#\#}\ p < 0.001$ *vs.* ***TDCNfs***, n=5.

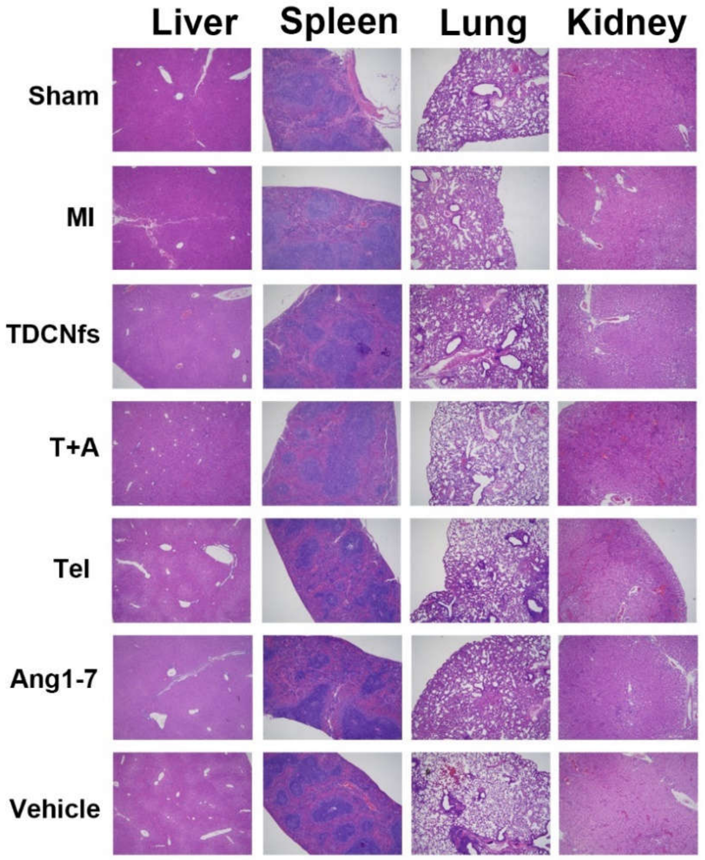

Figure S29. H&E staining of the major organs.

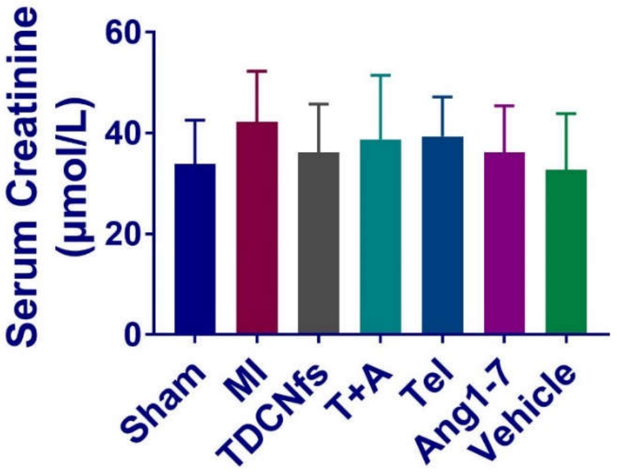

Figure S30. Serum expression of Cr level in different groups after 28 d treatment.

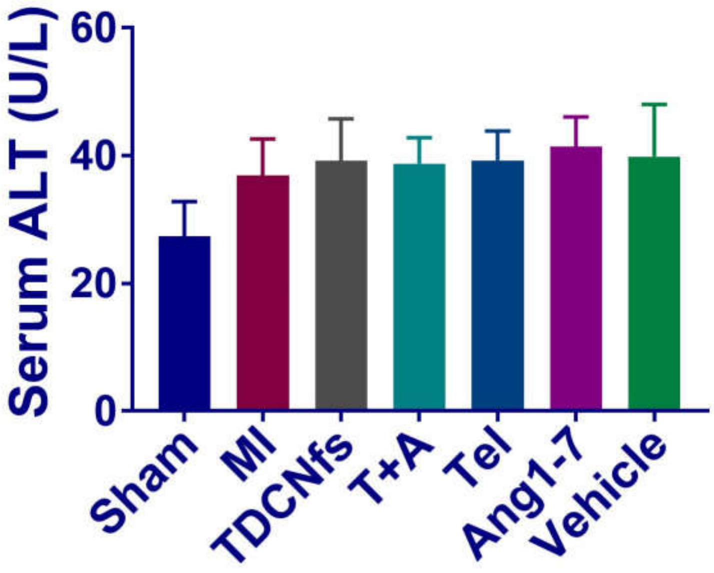

Figure S31. Serum expression of ALT level in different groups after 28 d treatment.

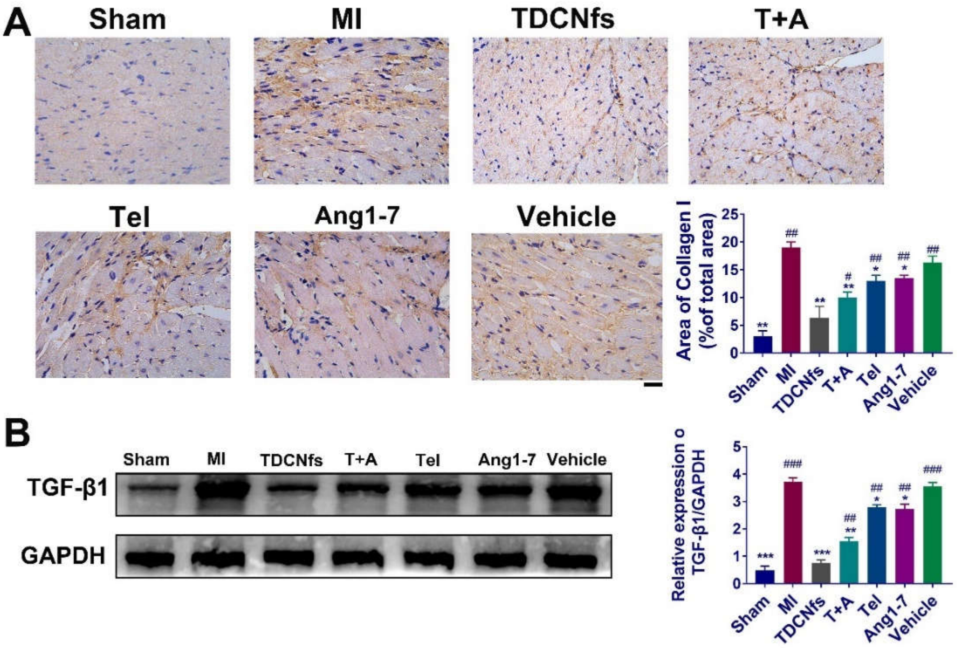

Figure S32. (A) Immunohistochemical staining of type I collagen in the non-infarcted regions of different treatment groups. Nucleus (blue), type I collagen (brown-yellow). Scale bar = 75 μm; Type I collagen quantified based on immunohistochemical area; (B) Western blot of TGFβ-1 and GAPDH serve as an internal reference; Quantification of bands using densitometry. $^{*}p < 0.05$ *vs.* MI, $^{**}p < 0.01$ *vs.* MI, $^{***}p < 0.001$ *vs.* MI, $^{\#}p <$ 0.05 *vs.* ***TDCNfs***, $^{\#\#}p < 0.01$ *vs.* ***TDCNfs*****,** $^{\#\#\#}p < 0.001$ *vs.* ***TDCNfs***, n = 5.

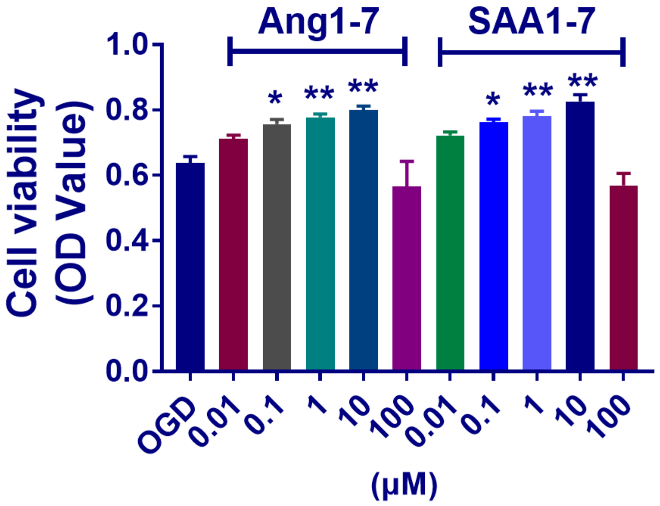

Figure S33. Cell viability of NRCMs cultured with ***SAA1-7*** and ***Ang1-7*** under OGD conditions ($^{*}p < 0.05$ *vs.* OGD group $^{**}p < 0.01$ *vs.* OGD group; n = 6 for each group); All data were obtained from three independent experiments.

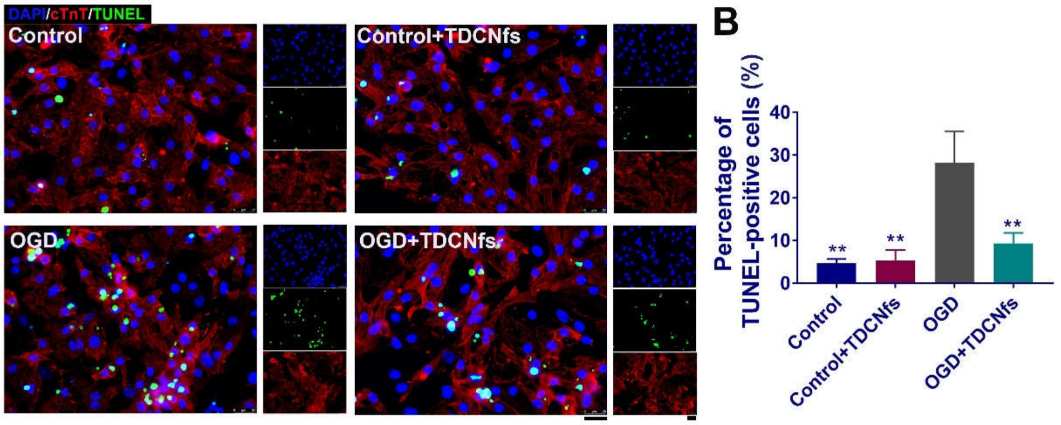

Figure S34. A) TUNEL staining of NRCMs in different groups; B) Quantitative analysis of TUNEL-positive NRCMs; Scale bar = 25 μm, $^{**}p < 0.01$ *vs.* OGD. n = 3.

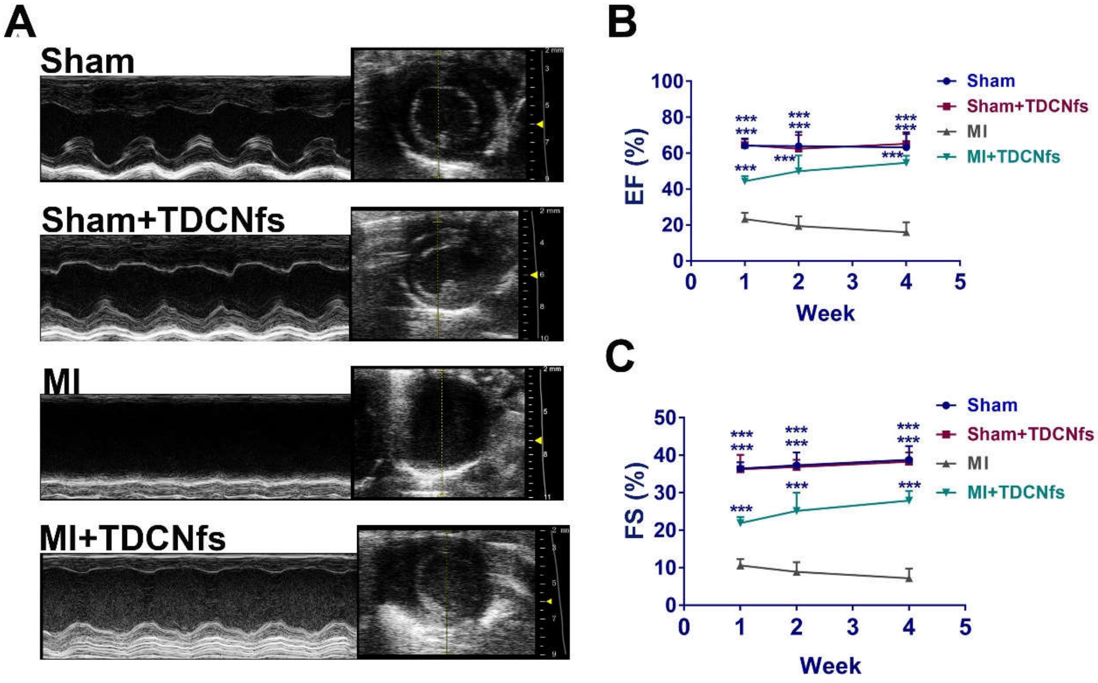

Figure S35. A) Representative echocardiograms (left) and measurements in different groups (1,2,4 weeks after LAD ligation); B) The percentage of left ventricular ejection fractions (EFs). C) The percentage of left ventricular fractional shortening (FS); $^{***}p < 0.001$ *vs.* MI, n = 5.

Table S1. Affinity study of ***SAA1-7*** and ARB obtained by Autodock 4.0 software.

Candidate Doped drugs	Binding energy (kcal/mol)	Ki (Mm)	Intermol. Energy (kcal/mol)	Internal Energy (kcal/mol)	Tors. Energy (kcal/mol)	Ref RMS
Losartan	-5.18	158	-7.87	-1.96	2.68	2.45
Valsartan	-4.22	800	-6.91	-2.18	2.68	2.62
**Telmisartan**	**-8.47**	**0.618**	**-9.66**	**-1.05**	**1.19**	**1.36**
Irbesartan	-7.24	4.9	-7.84	2.11	0.6	1.37
Candesartan	-5.29	131	-7.68	-2.1	2.39	1.48

Table S2. Drug formulations and doses

**Group**	**Drug**	**Dose**
Sham	PBS	100 μL/mouse
MI	PBS	100 μL/mouse
TDCNfs	NBD-^{D}F^{D}F^{D}Y^{D}E^{D}EG-DRVYIHP/Telmisartan	1084.16μg/kg,100 μL/mouse
T+A	Telmisartan+ DRVYIHP	328.96 μg/kg +576μg/kg,100 μL/mouse
Tel	Telmisartan	328.96 μg/kg 100 μL/mouse
Ang1-7	DRVYIHP	576μg/kg ,100 μL/mouse
Vehicle	NBD-^{D}F^{D}F^{D}Y^{D}E^{D}EG-YRVIPHD	1219.84μg/kg ,100 μL/mouse

Table S3. Primers sequences

Target genes	Forward primer sequence （5’-3’）	Reverse primer sequence （5’-3’）
Rat IL-1β	GCACTGCAGGCTTCGAGATGAAC	TTGGGATCCACACTCTCCAGCT
Rat IL-6	AGCCACTGCCTTCCCTACTTC	TGGTCTTGGTCCTTAGCCACTC
Rat TNFα	AGTCCGGGCAGGTCTACTTTG	CCACTACTTCAGCGTCTCGTGTG
Rat GAPDH	GGCAAGGTCATCCCAGAGCT	CCCAGGATGCCCTTTAGTGG

References

1. Li H, Gao J, Shang Y, Hua Y, Ye M, Yang Z, et al. Folic Acid Derived Hydrogel Enhances the Survival and Promotes Therapeutic Efficacy of iPS Cells for Acute Myocardial Infarction. Acs Appl Mater Inter. 2018; 10: 24459-68.
2. Shin M, Lee H, Lee M, Shin Y, Song J, Kang S, et al. Targeting protein and peptide therapeutics to the heart via tannic acid modification. Nat Biomed Eng. 2018; 2: 304-17.
